# Supplementary material for: An ultrasmall organic synapse for neuromorphic computing
Source: Nat Commun. 2023 Nov 23;14:7655. doi: 10.1038/s41467-023-43542-2 (PMC10667342; doi:10.1038/s41467-023-43542-2)
Supplement: Supplementary file 1 — Supplementary Informations.pdf [file 41467_2023_43542_MOESM1_ESM.pdf]

An ultrasmall organic synapse for neuromorphic computing

Shuzhi Liu[1,2†], Jianmin Zeng[1†], Zhixin Wu[1†], Han Hu[3†], Ao Xu[4], Xiaohe Huang[5], Weilin Chen[1], Qilai Chen[6], Zhe Yu[6], Yinyu Zhao[3], Rong Wang[3], Tingting Han[4], Chao Li[4], Pingqi Gao[6], Hyun-woo Kim[7], Seung Jae Baik[7], Ruoyu Zhang[3*], Zhang Zhang[4*], Peng Zhou[5*] and Gang Liu[1*]

[1]Department of Micro/Nano Electronics, School of Electronic Information and Electrical Engineering, Shanghai Jiao Tong University, Shanghai 200240, China.

[2]School of Chemistry and Chemical Engineering, Shanghai Jiao Tong University, Shanghai 200240, China.

[3]Ningbo Institute of Materials Technology and Engineering, Chinese Academy of Sciences, Ningbo 315201, China.

[4]School of Microelectronics, Hefei University of Technology, Hefei 230601, China.

[5]State Key Laboratory of ASIC and Systems, School of Microelectronics, Fudan University, Shanghai 200433, China.

[6]School of Materials, Sun Yat-Sen University, Guangzhou, Guangdong, 510275, China.

[7]School of Electronic and Electrical Engineering, Hankyong National University, Anseong-si, Gyeonggi-do, 17579, Korea

All correspondence and request for materials should be addressed to: zhangruoy@nimte.ac.cn (Prof. Ruoyu Zhang), zhangzhang@hfut.edu.cn (Prof. Zhang Zhang), pengzhou@fudan.edu.cn (Prof. Peng Zhou) and gang.liu@sjtu.edu.cn (Prof. Gang Liu)

[†]These authors contribute equally to this work.

Table of Contents

Section 1. Random evolution of ion migration-based CF in polymer synapses 3

Section 2. Synthesis of the polymer switching matrix PBFCL$_{10}$ 4

Section 3. Characterization of the polymer switching matrix PBFCL$_{10}$ 6

Section 4. Local switching characteristics and quantized conductance model 13

Section 5. Fabrication and switching mechanism of the PBFCL$_{10}$ devices 16

Section 6. Switching and integration performance of the PBFCL$_{10}$ devices 20

Section 7. Quantized conductance behavior of the PBFCL$_{10}$ devices 29

Section 8. Mixed-signal neuromorphic system based on the PBFCL$_{10}$ device 37

Section 9. Synaptic plasticities of the PBFCL$_{10}$ device 39

Section 10. Circuit design of the STDP comparator 46

Section 11. Operation of the bioinspired Hopfield neural network 50

Section 1. Random evolution of ion migration-based CF in polymer synapses

In organic semiconductors, the molecular level defects associated with the polymeric chain ends and folds, stacking faults, dislocations, as well as the macroscopic grain boundaries, voids and cracks, usually show low activation energy and serve as preferred migration sites for ionic species. Since most of the organic thin films are polycrystalline or amorphous with obvious structural inhomogeneity, the distribution of ion migration sites is therefore spatially uneven, giving rise to the formation of randomly distributed, highly localized and branched conductive nanofilaments (Supplementary Fig. 1a). Downscaled fabrication of memristive synapses onto samples with randomly distributed and evolved conductive filaments may results in the allocation of devices in regions that show dissimilar electrical characteristics or do not even exhibit any resistive switching behaviors at all (Supplementary Fig. 1b), making dimension shrinking into nanometer scale and integration into high-density matrix difficult for practical applications.

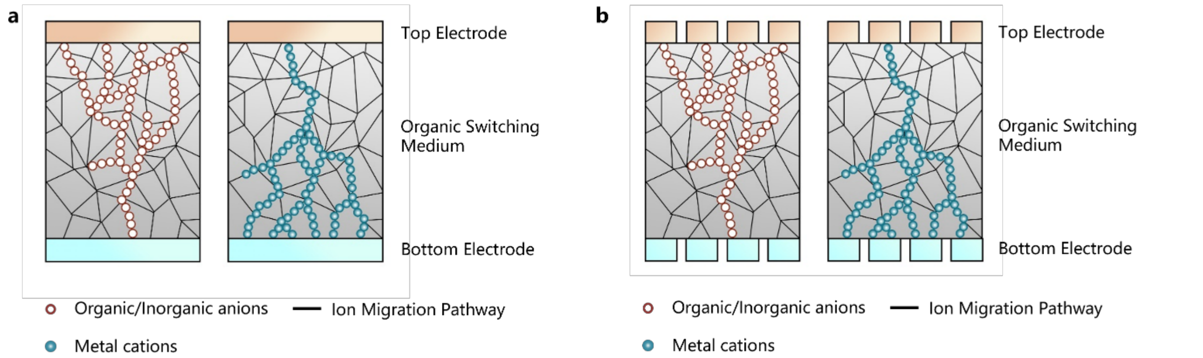

Supplementary Fig. 1 | Schematic illustration of the stochastic formation and evolution of conductive nanofilaments in structurally disordered organic material based synaptic devices with (a) large and (b) small sizes, respectively.

Section 2. Synthesis of the polymer switching matrix $PBFCL_{10}$

(1) Materials

Poly(ε-caprolactone) (PCL) diol oligomers (>99%，M_n=2000 g/mol), which are derivatives of natural bioproducts, were provided by Shenzhen Esun Industrial Co., Ltd. (China). Antioxidant 1010, 1,4-Butanediol (BDO), tetrabutyl titanate (TBT), 1,1,1,3,3,3-hexafluoro-2-propanol (HFP), methanol and antimony trioxide (Sb_2O_3) were purchased from Aladdin. 2,5-Furan dicarboxylic acid (FDCA, 99%) was purchased from Chem Target Technologies Co., Ltd. (China). The dimethyl furandicarboxylate (DMFD) was prepared based on FDCA[1]. Phenol and tetrachloroethane were also purchased from Aladdin as solvents for viscosity measurements.

(2) Synthesis of intermediate oligomer PBF

The synthesis routes of oligomers and polymers are displayed in Supplementary Fig. 2[2]. DMFD and BDO with the ratio of 1/1.3 (mol/mol) were added into a 1 L round-bottom flask equipped with a condenser, mechanical stirrer, collecting vessel and N_2 inlet. TBT (0.15 mol% of the DMFD) was added to facilitate the transesterification. The flask was repeatedly evacuated and purged by highly purified N_2 (99.999%) 3 times. Then the transesterification reaction was lasted at 170-200 °C for 4-6 hours, which could be considered to be finished when more than 95% theoretical methanol was obtained. The as-obtained product could be used directly as the hydroxyl terminated oligomer of poly(butylene furandicarboxylate) (PBF).

(3) Synthesis of target polymer $PBFCL_{10}$

Proper amounts of PBF oligomer and PCL diol were added into the reaction container. The targeted copolymer was defined as $PBFCL_{10}$, wherein the subscript 10 meant 10 mol % of CL

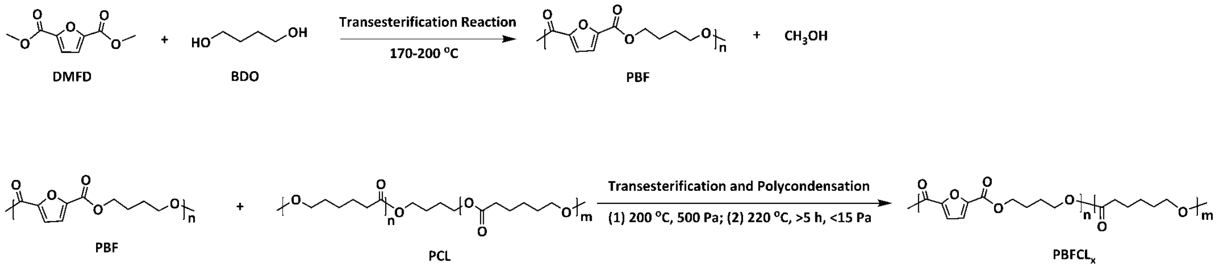

Supplementary Fig. 2 | Synthesis routes of PBF and $PBFCL_x$ from DMFD, 1,4-BDO, and PCL diol oligomers.

units in the copolyester. To protect the reaction and intermediate products, Sb_2O_3 (0.15 wt%) and antioxidant 1010 (0.1 wt%) were put into the reactor. The reaction system was kept at 500 Pa and 200 °C, and then it was further depressurized to less than 15 Pa during heating to 220 °C to minimize possible thermal degradation and oligomers sublimation. The progress of polymerization was evaluated by the torque value of the mechanical stirrer, as the viscosity of the system gradually increased with reaction time. The polycondensation reaction should be lasted for at least 5 h, so that sufficient transesterification between the two oligomers could be ensured. The stable torque value indicated the completion of the polycondensation reaction. Then the product was taken out under the protection of N_2, and cooled down to room temperature. The as-prepared $PBFCL_{10}$ was further purified to remove the residue prepolymers and monomers. The $PBFCL_{10}$ was dissolved in HFP and the solution was stirred at room temperature overnight. Then the solution was dropwise added into the stirring methanol. Such dissolution and precipitation processes were performed 3 times. At last, $PBFCL_{10}$ was dried in a vacuum oven for 72 h at 50 °C to completely remove the remaining methanol and HFP. The intrinsic viscosity of the as-obtained polymer was recorded with a mixed solvent of phenol and tetrachloroethane (1/1, w/w) in an Ubbelohde viscometer at 25 °C, at the concentration of 0.5 mg/mL.

Section 3. Characterization of the polymer switching matrix $PBFCL_{10}$

(1) Instruments

Molecular weights were determined with a Waters 2690 gel permeation chromatography (GPC) using polystyrene standards eluting with tetrahydrofuran. The intrinsic viscosity was recorded by an Ubbelohde viscometer. ^{1}H nuclear magnetic resonance (NMR) spectra were recorded on a Bruker 400 spectrometer at 400 MHz in deuterated chloroform with tetramethylsilane (TMS) as reference for the chemical shifts. Rheological analysis was conducted on DHR2 (TA Instruments). The basic thermal parameters were measured by DSC (Perkin Elmer Diamond DSC) with a heating/cooling rate of 10 °C/min. Grazing Incidence Wide Angle X-ray Scattering (GIWAXS) experiments were performed using a Xenocs Xeuss 3.0 beamline at the Vacuum Interconnected Nanotech Workstation (NANO-X) of Suzhou Institute of Nano-Tech and Nano-Bionics, Chinese Academy of Sciences. The Xeuss 3.0 (GI-)SAXS/WAXS/USAXS beamline was equipped with dual X-ray sources, an Excillum high flux gallium metal jet source and a Xenocs GeniX^{3D} micro-focus copper source. An X-ray wavelength of λ = 1.3414 Å and a beam size of (0.9×0.9) mm^2 were employed for the measurements. The scattered intensities were collected using a Dectris EIGER2 R 1M detector at a sample to detector distance of 130 mm, with an instrument resolution of ~0.02 Å^{-1}. GIWAXS data were collected with an exposure time of 1200 s and X-ray incident angle of 0.2°. X-ray diffraction (XRD) patterns were obtained on Bruker AXS D8 Advance using Cu-Kα radiation. Scanning electron microscopic observation was conducted on a RISE-MAGNA system.

(2) Molecular weight, composition and viscosity

The successful synthesis of $PBFCL_{10}$ with an average molecular weight (M_w) of 67.9 kDa,

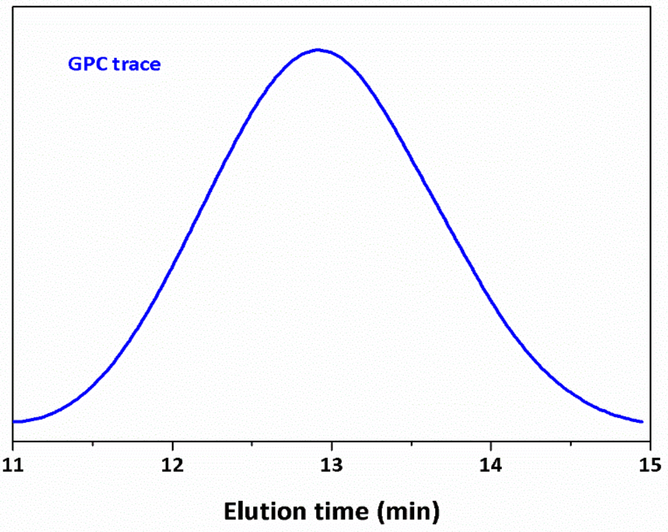

Supplementary Fig. 3 | Gel permeation chromatography (GPC) curves of $PBFCL_{10}$.

polydispersity index (PDI) of 1.8 and intrinsic viscosity of 1.1 dL/g was verified by the chromatography and viscosity analysis (Supplementary Fig. 3). The high molecular weight and good solubility of the as-synthesized macromolecules in chloroform allow great solution processability for obtaining dense and uniform polymer thin films. The composition, sequence length and probability of dyads in $PBFCL_{10}$ can be calculated with the ^{1}H NMR data as shown in Supplementary Fig. 4 by the following equation,

$$\Phi_F = \frac{I_a}{I_a+I_h} \quad (1)$$

$$\Phi_{CL} = \frac{I_h}{I_a+I_h} \quad (2)$$

$$L_F = \frac{2\Phi_F}{\Phi_{FCL}} = \frac{2I_a}{I_c+I_d} \quad (3)$$

$$L_{CL} = \frac{2\Phi_{CL}}{\Phi_{FCL}} = \frac{2I_h}{I_c+I_d} \quad (4)$$

$$P_{FCL} = \frac{\Phi_{FCL}}{2\Phi_F} = \frac{1}{L_F} \quad (5)$$

$$P_{CLF} = \frac{\Phi_{FCL}}{2\Phi_{CL}} = \frac{1}{L_{CL}} \quad (6)$$

where I_a and I_h belong to the integral intensity of ^{1}H at the β-position of the furan ring and α-

position of the ester group from the PCL monomer, respectively (Equations 1 and 2). Φ_F and Φ_{CL} ascribe to the content of BF and CL unit in $PBFCL_x$, respectively. Φ_{FCL} is the sum of the integral intensity of ^1H at peaks c and d, which are attributed to the $-CH_2-$ group that construct the passage between furan rings. L_F and L_{CL} are the number-average lengths of the BF and CL blocks of the copolymer, and could be obtained from the integral intensity of I_a and I_h through Equations 3 and 4, respectively. P_{FCL} is the probability of a given CL unit with a BF unit on the right, while P_{CLF} is the probability of a given BF unit with a CL unit on the right, both of which can be calculated by Equations 5 and 6, respectively. By virtue of peak assignment in the ^1H NMR spectrum, $PBFCL_{10}$ has an exact composition ($\Phi_{CL, \% mol}$) of 13.7% for the CL segments that is slightly higher than the nominal value but is still experimentally acceptable (Supplementary Table 1). The number-average lengths (L_F and L_{CL}) of the BF and CL blocks in the copolymers are 7.6 and 1.2, while the probability of finding a CL monomer unit next to a BF monomer unit (P_{CLF} and P_{FCL}) are 0.833 and 0.132, respectively. Such molecular structure information indicates that each flexible blocks of $PBFCL_{10}$ carry no more than two monomer units of CL, which therefore only acts as gluing agents to connect the adjacent rigid furan-containing BF blocks and make the polymer soluble in the meantime. The small size and minor content of the CL blocks also avoid the occurrence of room-temperature phase separation of the originally incompatible BF and CL components.

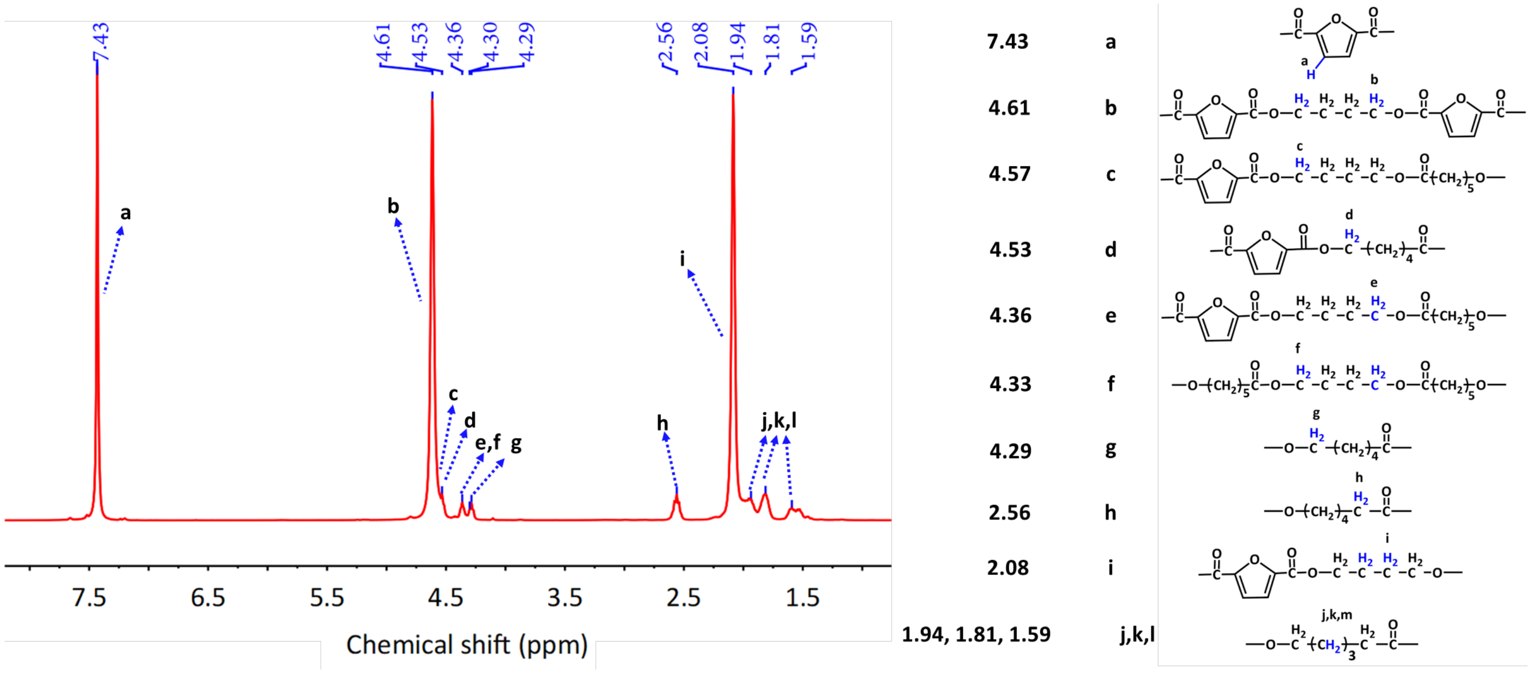

Supplementary Fig. 4 | ^{1}H NMR spectrum and peak assignments of $PBFCL_{10}$.

Supplementary Table 1. The molecular characteristics of $PBFCL_{10}$

Sample	Composition	Sequence Length		Probability of Dyads		Intrinsic Viscosity	GPC	
	Φ_{CL} *(mol %)*	L_F	L_{CL}	P_{CLF}	P_{FCL}	$[\eta]$	M_w	*Đ*
$PBFCL_{10}$	13.7	7.6	1.2	0.833	0.132	1.10	$6.79*10^4$	1.8

(3) Structural properties

As plotted in Supplementary Fig. 5, the pseudo constant slop of the frequency-dependent storage modulus and single arc shape of the Cole-Cole curves at low temperatures suggest that micro-phase separation in $PBFCL_{10}$ only occurs when being heated over 180 ºC. Note that the state-of-the-art microelectronic devices and integrated circuits generally work at temperatures not higher than 125 ºC, single-phase material with spatially homogeneous properties will be safely maintained in the $PBFCL_{10}$ thin film and make them thermodynamically stable during synapse device operations.

Calorimetric analysis indicates that the pristine $PBFCL_{10}$ has glass transition, cold crystallization, melting and recrystallization temperatures of 28.4 ºC, 81.1 ºC, 159.5 ºC and 100.4 ºC, respectively (Supplementary Fig. 6). Thermal treatments between 100 ºC and 160 ºC will therefore generate a certain degree of microcrystallinity inside the thin films of the copolymer. Grazing incident wide-angle X-ray scattering (GIWAXS) measurement reveals that the spin-coated and thermally annealed $PBFCL_{10}$ thin film shows a semicrystalline nature (Fig. 1b and Supplem

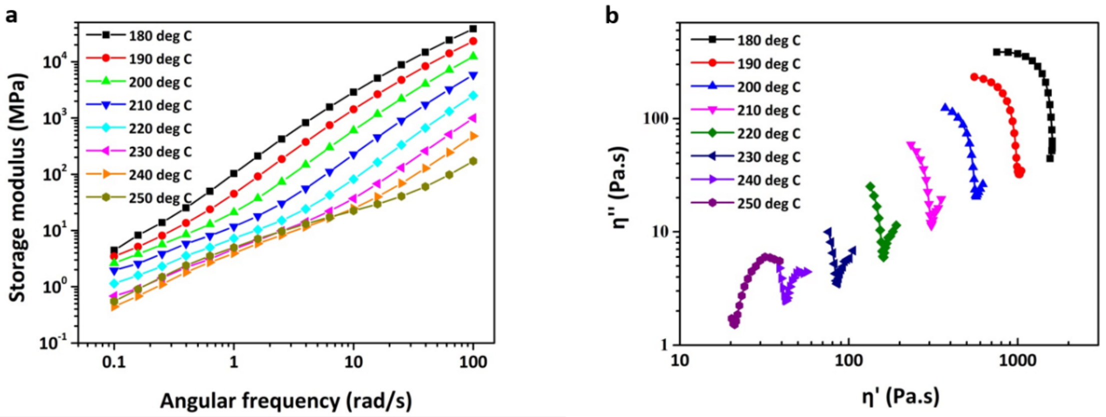

Supplementary Fig. 5│ (a) The frequency dependent storage modulus and (b) Cole-Cole curves of $PBFCL_{10}$ recorded at different temperatures.

entary Fig. 7). The isotropic scattering ring can be ascribed to the macroscopically in-plane vortex alignment of the lamellar crystals' (001) lattice plane at the 18.3° diffractive angle 2θ of the furan rigid segments (Supplementary Fig. 8a)[2], while the arc signals at the q_z of 1.73 Å^{-1} and 2θ of 21.3° are associated with the ordered alignment of (010) lattice plane in the out-of-plane direction. Although spin-coating is a thermodynamically metastable process, in which the fast evaporation of the solvent molecules does not allow enough time for a complete crystallization of the polymer material, the centrifugal tendency of the spinning procedure still provides driving force for the $PBFCL_{10}$ crystallites to align in an isotropically ordered manner. Supplementary Fig. 8b shows the furan segment based lamellar crystals with in-plane vortex distribution and out-of-plane anisotropic alignment in the spin-coated $PBFCL_{10}$ thin film.

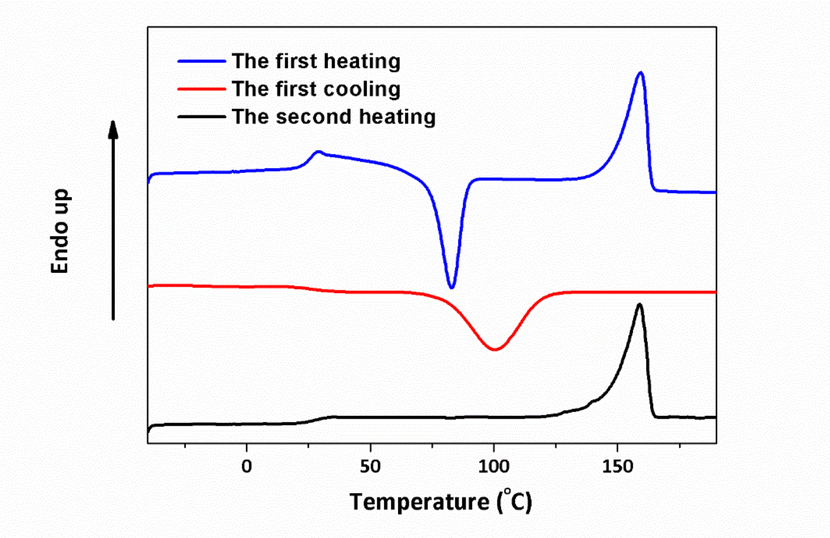

Supplementary Fig. 6 | The differential scanning calorimetry (DSC) scan curves of $PBFCL_{10}$ in the first heating, cooling and the second heating procedures, respectively.

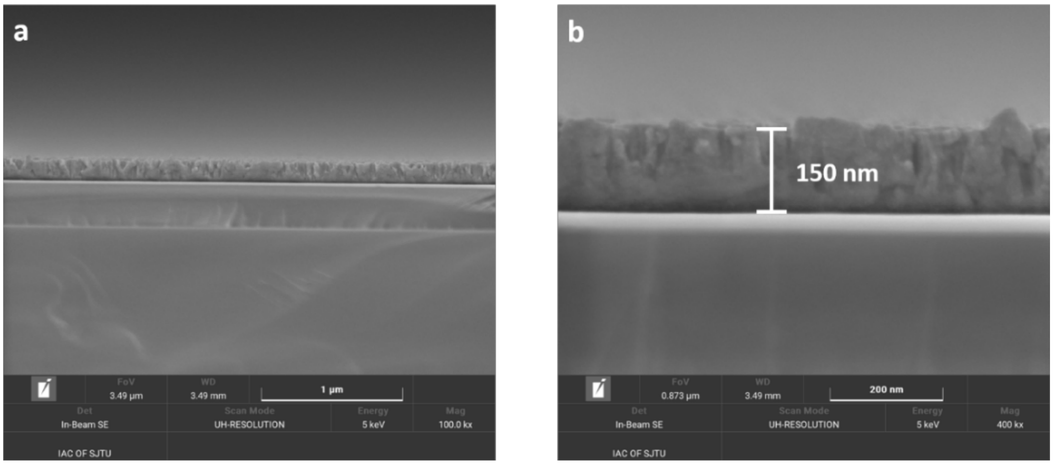

Supplementary Fig. 7│ Cross-sectional scanning electron microscope (SEM) images of the $PBFCL_{10}$ film visualized at different magnifications.

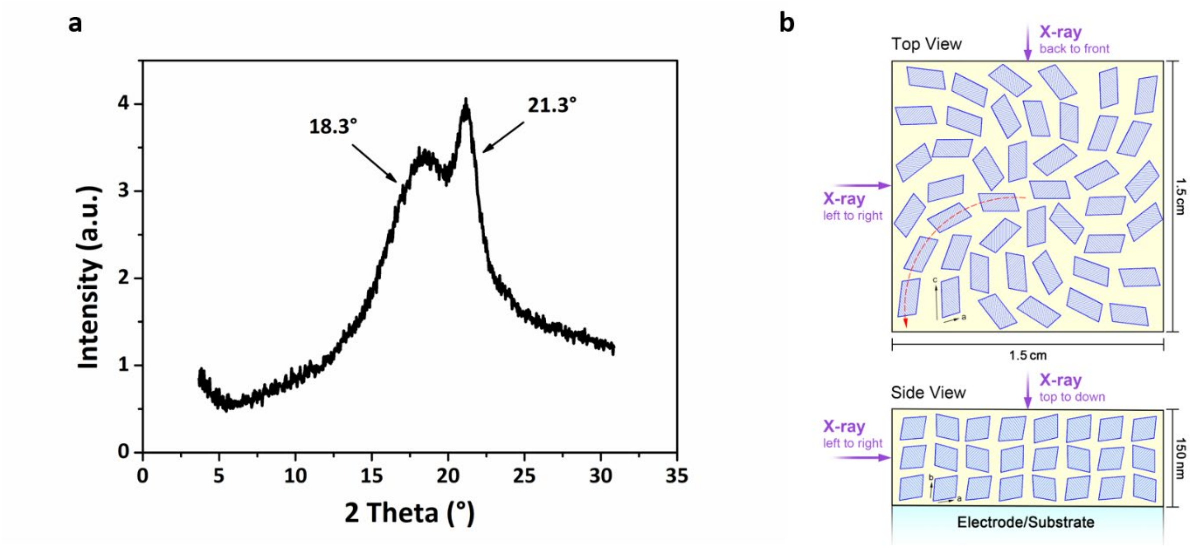

Supplementary Fig. 8│ (a) 1D GIWAXS profile of $PBFCL_{10}$ along the longitude direction. (b) Schematic illustration of the furan segment based lamellar crystals with macroscopically in-plane vortex distribution and out-of-plane anisotropic alignment in the spin-coated $PBFCL_{10}$ thin film. For better illustration, the lamellar crystals in b are not drawn to scale.

Section 4. Local switching characteristics and quantized conductance model

(1) Methods

Atomic force microscopy (AFM) and conductive-atomic force microscopy (C-AFM) measurements were performed on a Solver P47-PRO (NT-MDT Co., Moscow, Russia) microscope to monitor the surface morphology and local conduction behavior of the polymer thin film deposited on the Ag coated SiO_2/Si substrate. During C-AFM measurements, a conducting cantilever coated with Pt was used as movable and grounded top electrode to form a Pt/$PBFCL_{10}$/Ag structure, while the voltage pulses were applied through the universal bottom silver electrode to the polymer thin film. Upon scanning across the $PBFCL_{10}$ layer with the Pt-coated cantilever, the spatial distribution and amplitudes of the currents recorded in the C-AFM current profile can be used to semi-quantitatively reflect the corresponding distribution and sizes of the local conductive regions or conductive nanofilaments in the scanned area.

(2) Local switching characteristics

The as-deposited $PBFCL_{10}$ layer was in the high resistance state initially. Then a 1 V voltage stimulus was applied through the bottom electrode, and the polymer layer was scanned over a 1 μm^2 area. Afterwards, the amplitude of the applied voltage was decreased to 0.1 V to read the current response of the sample. As shown in the right panel of Fig. 1e, dense and uniform distribution of conductive nanofilaments with the inter-CF spacing of 40 nm ~ 50 nm appeared, suggesting that the $PBFCL_{10}$ layer had been switched to ON state. Similarly, as depicted in the left panel of Fig 1e, the C-AFM current map of a 50 nm×50 nm region showed insulating behavior with tiny leakage current of ~ 5.2 pA in its as-fabricated OFF state. When the area was scanned with the C-AFM tip and 1 V voltage through the Ag electrode, an obvious conductive

region with the diameter of 43.5 nm appeared when read with a 0.1 V voltage. As depicted by the height of the recorded peak, the largest current value of the conductive region is 33.4 nA. It corresponded to the formation of one single conductive nanofilament that switched the scanned area to a low resistance state (LRS, or ON state) with an ON/OFF ratio of 6.4×10^3. When voltage stimuli of -0.5 V, -1.0 V and -1.5 V were applied to the scanning area consecutively, the peak currents and diameters of the conductive region read at 0.1 V decreased to 24.1 nA/34.7 nm, 10.8 nA/23.8 nm and 6.7 nA/11.8 nm, respectively. This is in good accordance with the negative feedback mechanism that gradually modulates the size of the conductive filament and conductance of the memristive synapse with decreasing device currents. It provides the possibility of forming APC in the connecting-disconnecting critical state of the conductive filament. Note that the resolution of the C-AFM measurement is 256 pixel/1μm, which equals to 3.91 nm/pixel. Therefore, the observation of conductive filament with the size of a few nanometers can be considered faithfully through C-AFM measurements.

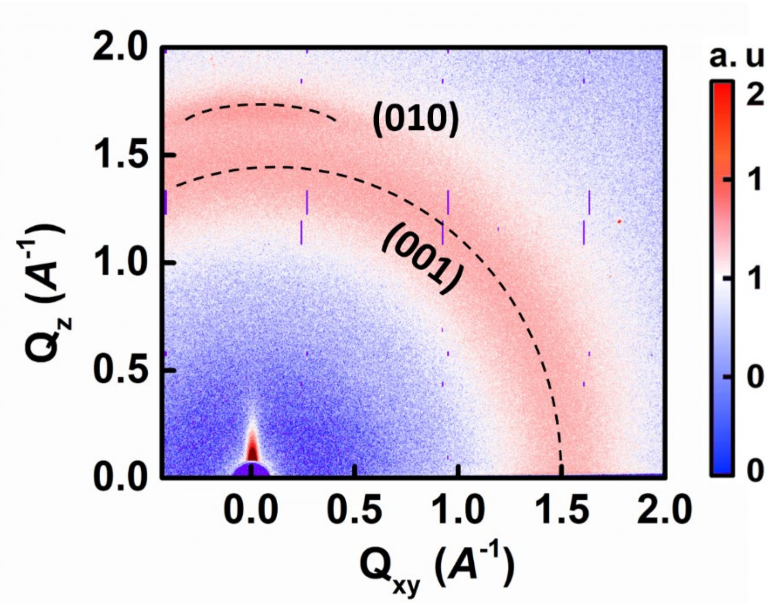

Supplementary Fig. 9│ GIWAXS pattern of $PBFCL_{10}$ film after SET and RESET operations.

To evident the structural stability of the polymer layer, we re-conduct GIWAXS measurement after the C-AFM based SET/RESET operations on the $PBFCL_{10}$ film. As displayed in Supplementary Fig. 9, the scattering pattern of the $PBFCL_{10}$ film shows similar crystal assignment and intensity after the formation of conductive filaments. It suggests that although the formation of CFs may expand the grain boundaries slightly, the crystallinity and orientations of the polymer crystals are well maintained. Therefore, the structural stability of the semicrystalline $PFBCL_{10}$ endows itself great potential for practical neuromorphic device applications.

(3) Quantized conductance model

AFM observation reveals that the diameter of the polymer lamellae in the $PBFCL_{10}$ film is ~ 45 nm. In accordance with the hypothesis that ion migration and CF formation occur along the grain boundaries, as well as the 45 nm diameter of the polymer fibrillar lamellae, C-AFM observation reveals that the inter-spacing between the neighboring CFs is ~ 40 nm to 50 nm. As illustrated in Supplementary Fig. 10, when the organic synapse size scales down to 50 nm,

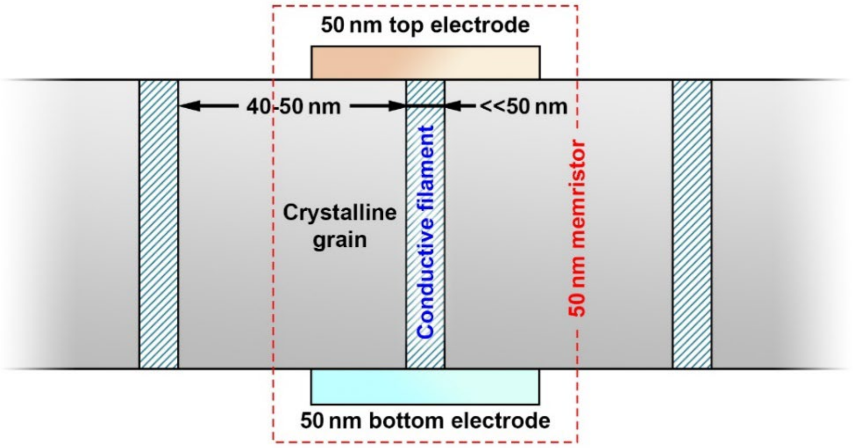

Supplementary Fig. 10 | Schematic illustration of relationship between the size of the conductive filament, the size of the crystalline grains and the 50 nm $PBFCL_{10}$ device.

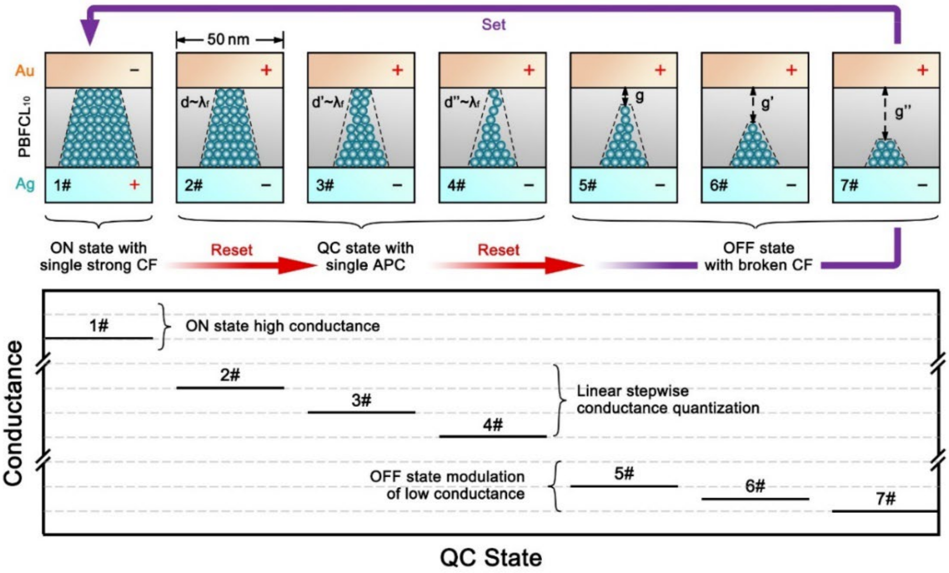

Supplementary Fig. 11│ Illustration of the single conductive nanofilament and atomic point contact evolution in the 50 nm $PBFCL_{10}$ synapse device.

no more than one grain boundary will exist in the device. Consequently, only one conductive filament will be formed in the 50 nm size $PBFCL_{10}$ device, the fine-tuning of which will lead to the formation of atomic point contact showing conductance quantization characteristics.

In atomic point contact structures, as the lateral dimension of the nanofilament (diameter at the narrowest end, in particular) shrinks to that comparable to the Fermi wavelength or mean free path of electrons (Supplementary Fig. 11), charge carriers will move through the quasi-one-dimensional electron system ballistically without scattering[3-5]. The device conductance in consequence closely depends on the size of the atomic point contact, and is quantized in the half-integer unit of $G_0 = 2e^2/h$ = 77.5 uS (where e is the elemental charge of electrons and h is the Planck's constant). Such dimension-related multilevel conductance quantization behavior may provide an intrinsic platform of the linear synaptic weight updating required during neuromorphic computing iterations.

Section 5. Fabrication and switching mechanism of the $PBFCL_{10}$ devices

(1) Fabrication of the $PBFCL_{10}$ synapse device

The Au/$PBFCL_{10}$/Ag structure devices were fabricated through electron beam lithography technique and a lift-off approach. Crossbar electrode strips with a width of 50 nm and a separation of 85 nm between each other were formed on a SiO_2/Si substrate. The 32 bottom electrode strips were patterned by electron beam lithography with a Vistec EBPG-5200$^+$ Electron-beam lithography system and E-beam evaporation of a 40 nm Ag layer on top of a 5 nm Ti adhesion layer with a Denton Electron Beam Evaporator. After lift-off, 30 μL of $PBFCL_{10}$ solution (5 mg/mL) in chloroform was spin-coated onto the bottom electrodes at 3000 rpm for 60 s. Afterward, the obtained samples were dried at 60 °C in vacuum for 6 h to form a continuous polymer layer on top of the bottom electrodes and the substrate. Finally, 32 top electrode stripes consisting of 5 nm Ti and 40 nm Au were patterned and deposited using electron beam lithography, e-beam evaporation, and lift-off process similarly. Being consistent with that of the bottom electrodes, the linewidth and separation of the top electrodes are 50 nm and 85 nm, respectively. Therefore, Au/$PBFCL_{10}$/Ag synapse devices are formed at the cross points of the orthogonally aligned top and bottom electrodes. Note that the polymer switching layer was not patterned in the present study, the dimension of the synapses is defined by the linewidths of the electrode strips.

(2) Switching mechanism of the $PBFCL_{10}$ synapse device

UV-visible absorption spectral measurements were conducted on a Lamda 950 spectrophotometer to study the ion migration and switching mechanism of the $PBFCL_{10}$ based synapse devices. To perform the measurements, the stock solution of $PBFCL_{10}$ with the concentration

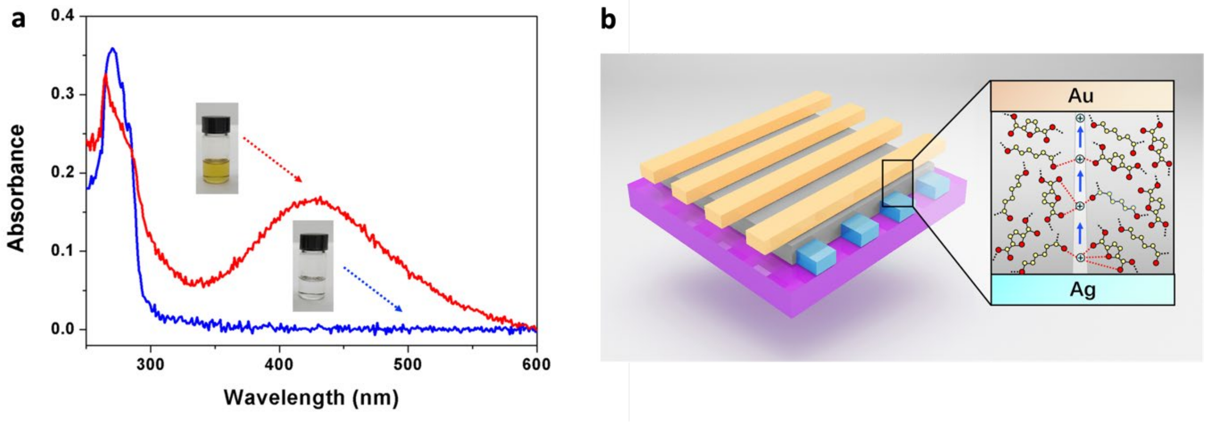

Supplementary Fig. 12│ (a) UV-visible absorption spectra of the $PBFCL_{10}$ chloroform solutions before (blue) and after (red) the addition of silver ions, respectively. Inset shows the photographs of the polymer solution samples before and after adding silver ions, respectively. (b) Schematic illustration of Ag^{+} ion migration and formation of conductive nanofilament inside the Au/$PBFCL_{10}$/Ag structured synapse device.

of 1 mg/ml was prepared in chloroform and the stock solution of silver nitrate with the concentration of 2 mg/ml in was prepared DMF. Both silver nitrate and *N*,*N*-dimethylformamide (DMF) were purchased from Aladdin. Afterwards, the blank control sample was prepared by diluting 10 μL of the $PBFCL_{10}$ stock solution into a 10 ml solution in a mixed solvent of DMF/chloroform (1:1 = v:v). The test sample was prepared by combining 20 μL of the silver nitrate stock solution and 10 μL of the $PBFCL_{10}$ stock solution, and diluted to a 10 ml solution in a mixed solvents of DMF/chloroform (1:1 = v:v). As shown in the photographs and UV-visible absorption spectra of the Supplementary Fig. 12a, mixing $PBFCL_{10}$ with silver ion will turn the solution from colorless into light yellow with a newly appearing mild absorption band in the 350 nm to 600 nm region, which suggests the effect coordination of Ag^{+} species with the oxygen-containing function groups of the polymer. In the solid-state layer, the coordination of the Ag^{+} ions with the polymer matrix, their movement from one position to another through the mild

wriggle of the flexible CL chains, discoordination from the present chelating sites and further coordination with the next oxygen-containing moieties enable the cation migration across the polymer thin film.

Supplementary Fig. 12b depicts the coordination of Ag ions with $PBFCL_{10}$ and switching mechanism of the Au/polymer/Ag synapse device (similar to that of the Pt/$PBFCL_{10}$/Ag structure during C-AFM measurement). It should be noted that during operation all the voltage stimuli were applied onto the bottom Ag electrodes while the top Au electrodes (or the Pt/Ir-coated conducting AFM tips) were always grounded. When the biased voltage was applied between two electrodes, the Ag bottom electrode undergoes oxidation ($Ag - e^- \rightarrow Ag^+$) and ion injection into the $PBFCL_{10}$ film. The injected Ag ions subsequently coordinate with the oxygen-containing moieties ($nAg^+ + PBFCL_{10} \rightarrow [Ag\text{-}PBFCL_{10}]^{n+}$) and migrate across the $PBFCL_{10}$ layer through mild molecular motions of the soft CL segments. During the electric field-driven migration, the silver ions receive electrons from the Au cathode and get reduced to their neutral atomic form ($[Ag\text{-}PBFCL_{10}]^{n+} + ne^- \rightarrow nAg + PBFCL_{10}$). These Ag atoms aggregate into cluster and extend to the Au cathode, which finally lead to the formation of an Ag conductive nanofilament that connects the top and bottom electrodes and switch the device from the initial high resistance OFF state to the low resistance ON state[6].

Cross-sectional transmission electron microscope (TEM) was also conducted to visualize the formation of single Ag filament in the 50 nm $PBFCL_{10}$ synapse device. Herein, a $PBFCL_{10}$ device in the 32×32 crossbar array was firstly switched to the ON state by a 5 V voltage stimulus. Then the cross-sectional specimen was prepared with the ON-state device using focus-ion beam

technique. As indicated by the energy dispersive spectral (EDS) profile of Supplementary Fig. 13, a weak orange region can be seen dimly inside the polymer layer between the top Au/Ti (red/green) and bottom Ag (orange) electrodes. It indicates that a single Ag filament with the size of 5 nm ~ 10 nm is formed in the 50 nm $PBFCL_{10}$ synapse device.

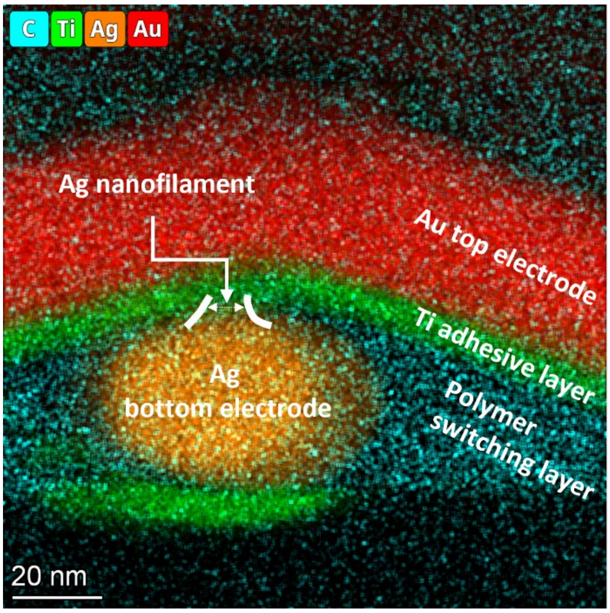

Supplementary Fig. 13│ Energy dispersive spectral mode cross-sectional TEM image of the ON-state Au/Ti/$PBFCL_{10}$/Ag/Ti device.

Section 6. Switching and integration performance of the $PBFCL_{10}$ devices

(1) Switching speed of the $PBFCL_{10}$ devices

The as-fabricated Au/$PBFCL_{10}$/Ag synapse is in the high resistance state, with the device conductance being ~ 18.6 nS when read at 0.1 V (Supplementary Fig. 14a). Upon direct-current voltage sweeping from 0 V to 5.5 V, an abrupt increase in the device current from 2.4×10^{-7} A to the compliance preset of 1.0×10^{-3} A occurs at the threshold voltage of 5.0 V. It corresponds to the formation of the silver conductive nanofilament that connects the top and bottom electrode, and resistive switching of the device from the initial resistance state to the low resistance ON state. Subsequent scanning in the negatively biased voltage sweep can switch the organic device back to a high resistance (OFF) state upon Joule heating assisted annihilation of the nanofilament. Afterwards, sweepings between ±1.0 V can switch the device ON and OFF repeatedly. As shown in Fig. 2b, at the threshold voltage of 0.2 V, the current of the $PBFCL_{10}$ device increases from 3.1×10^{-5} A to the compliance preset of 1.0×10^{-3} A and switches the device ON. Note that the OFF state and compliance currents of 3.1×10^{-5} A and 1.0×10^{-3} A herein are read at the threshold voltage of 0.2 V, which are the transient response of the device currents upon the occurrence of resistive switching in $PBFCL_{10}$ device. During the following negative sweeping, the device returns to the OFF state at -0.2 V, which completes the programing-erasing cycle of a memristive synapse. Due to the incomplete dissolution of the Ag nanofilament, the 116.5 μS conductance of the OFF state $PBFCL_{10}$ synapse (read at 0.1 V) is much higher than the initial value in the as-fabricated device.

The switching speed of the $PBFCL_{10}$ synapse was measured by monitoring the transient current responses upon the application of a 2.5 V constant voltage stimulus, as shown in Fig.

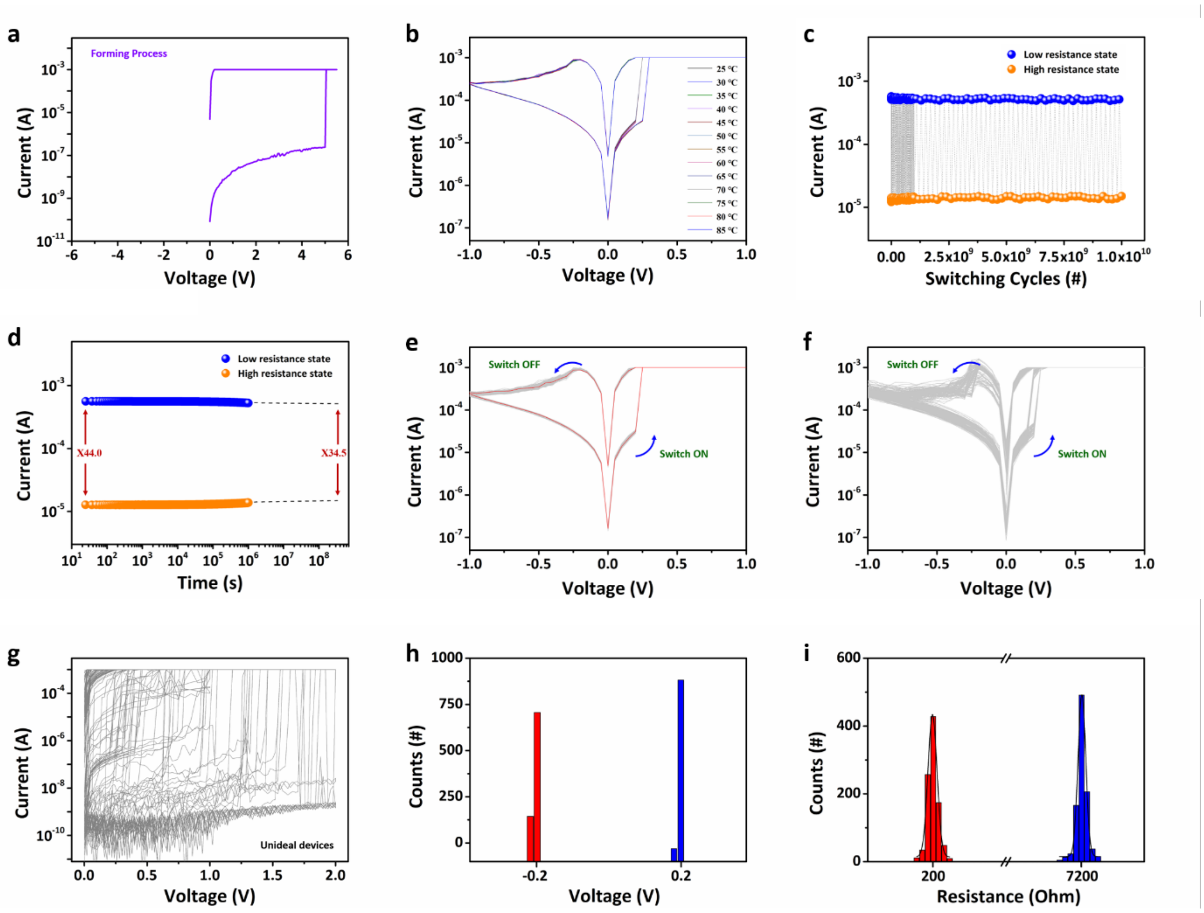

Supplementary Fig. 14| (a) Room-temperature current-voltage characteristic of the Au/PBFCL10/Ag device in the forming process. (b) Current-voltage characteristics of the Au/PBFCL10/Ag device recorded in the temperature range of 25 °C to 85 °C. (c) Endurance and (d) retention performance of the Au/PBFCL10/Ag device recorded at the temperature of 85 °C. All the device currents are read at 0.1 V. Room-temperature current-voltage characteristics of (e) 824 Au/PBFCL10/Ag devices showing repeatable bistable switching characteristics, (f) 123 Au/PBFCL10/Ag devices showing similar electrical performance but with minor fluctuations in the switching parameters, and (g) 77 remaining Au/PBFCL10/Ag devices in the 32×32 crossbar array. Distribution of (h) the Reset/Reset voltages and (i) ON/OFF state currents of the 947 stable-switching PBFCL10 devices. The data sets in (h) and (i) are extracted from the I-V curves of (e) and (f).

2c. Before measurement, the organic device was reset to the state with high resistance of ~ 4.30 kΩ. Then constant voltage stimulus of 2.5 V was introduced onto the device at t = 185 ns, following which a clear climbing and plateau period until 235 ns can be observed. The device resistance during the plateau period, read as 4.25 kΩ ~ 4.31 kΩ, is similar to that recorded

immediately after the RESET operation. Thus, the climbing and plateau period can be ascribed to the RC delay of the measuring system. The device resistance drops abruptly between 245 ns and 266 ns to the low level of 101.5 Ω, suggesting that the organic synapse is switched to the ON state during this period. Therefore, the OFF-to-ON transition time of the $PBFCL_{10}$ device under the voltage of 2.5 V is estimated as 21 ns. The energy consumption during this period can be calculated as 143.27 pJ, by integrating the transient curve shown in Fig. 2c according to $E = \int_0^T V_t^2 G_t \, dt$, where E is the energy consumption, V_t is the voltage applied onto the device at time t while G_t is the device conductance recorded at time t. Considering that RC delay always exists for capacitor like devices, another 55 ns should be added to count the nominal switching speed of the $PBFCL_{10}$ synapse, while the energy consumption increases to 186.39 pJ, respectively. In case that further approach can be developed to shorten the RC delay time in future work, both the switching speed and energy consumption of the organic neuromorphic device can be decreased significantly. Although the energy consumption of the present organic synapse is still larger than that of the devices based on volatile charge carrier hopping mechanism[7], it is

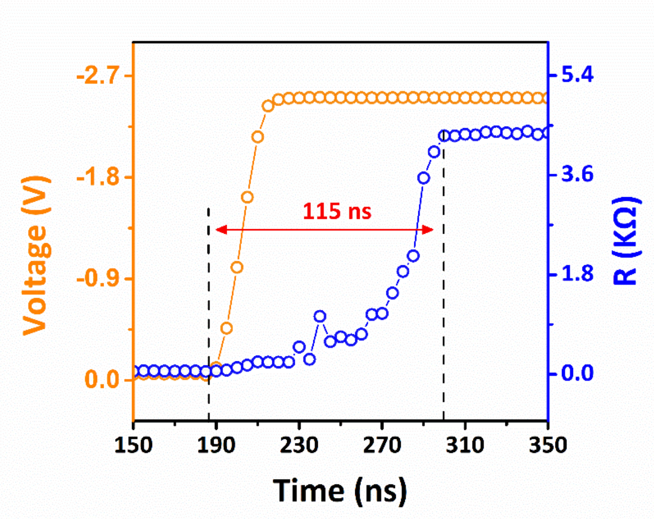

Supplementary Fig. 15| Device resistance in immediate response to an applied voltage stimulus of -2.5 V recorded during the reset process.

good enough for low power neuromorphic computing applications. Supplementary Fig. 15 displays the transient current response of the $PBFCL_{10}$ synapse recorded during reset process. Initially, the device was programmed to the ON state with a low-level resistance of 74.8 Ω read at -0.05 V. When a voltage stress with the amplitude of -2.5 V was applied onto the device, a gradual increase of device resistance to 4.34 kΩ can be observed. Therefore, a 115 ns ON-to-OFF switching speed of the $PBFCL_{10}$ synapse, including the RC delay time, can be read.

(2) Switching reliability of single $PBFCL_{10}$ synapse

Supplementary Fig. 14b plots the bistable switching characteristics of a $PBFCL_{10}$ device recorded in the temperature range of 25 °C to 85 °C with a ramping step of 5 °C. As shown, the Set/Reset voltages and ON/OFF state currents of the organic device (read at 0.1 V) remains almost the same when the temperature increases from 25 °C to 85 °C. According to

$$\text{Variation (\%)} = \delta/\mu \times 100\% \tag{7}$$

where δ and μ are the standard deviation and average value of the device parameters, the variation of the Set/Reset voltages and ON/OFF state currents recorded between 25 °C and 85 °C are derived as 10.36%, 6.80%, 1.14% and 7.80%, respectively. Supplementary Fig. 14c and 14d depict the evolution of the device currents, recorded at 85 °C and 0.1 V, during the endurance test of 10^{10} continuous switching cycles and retention test over 10^6 s. Endurance test was conducted in pulse mode, wherein the amplitudes and widths of the voltage pulses to switch the organic synapse are ±2.5 V and 100 μs. According to the above equation (7), the variations of the ON/OFF states currents during the endurance and retention tests are 3.76% (ON current of endurance test), 5.38% (OFF current of endurance test), 1.66% (ON current of retention test) and 1.25% (OFF current of retention test), respectively. As shown in Supplementary Fig. 14d,

linear extrapolation of the retention curve reveals that after ten years operation the ON/OFF ratio remains 34.5, which is estimated at the time of 3.15×10^8 s.

(3) Switching reliability of $PBFCL_{10}$ synapse crossbar array

Table 3 summarizes the device size, crossbar dimension and conductance uniformity of $PBFCL_{10}$ based synapse and other organic memristors recorded in the literatures[8-40]. As can be seen, the present $PBFCL_{10}$ device shows the smallest cell size of 50 nm and largest crossbar array dimension of 1 Kb among all the organic memristors reported so far. Supplementary Fig. 14e shows the room-temperature current-voltage characteristics of 824 working devices that exhibit repeatable bistable switching characteristics in the 32×32 $PBFCL_{10}$ synapse crossbar array, wherein each curve of a complete "write-erase" cycle is obtained from an individual device. The red trace shows a typical I-V curve of a $PBFCL_{10}$ device exhibiting bistable switching characteristics, while the grey traces show the I-V curves of the other 823 working devices in the array. Supplementary Fig. 14f plots the I-V curves of another 123 devices in the same array, which exhibit similar bistable switching behavior but with minor fluctuations in the programming voltages and ON/OFF state currents. Supplementary Fig. 14g depicts the I-V curves of the remaining 77 devices in the crossbar array. By extracting the programming voltages and ON/OFF state currents read at 0.1 V from Supplementary Fig. 14e and 14f, device-to-device (D2D) variations of the Set/Reset voltages and ON/OFF state resistances of the 947 working devices are estimated as 6.13%, 9.95%, 3.25% and 6.66% (Supplementary Fig. 14h and 14i), respectively, according to the above equation (7).

Supplementary Table 2. Set/Reset voltages and ON/OFF state currents of the Au/$PBFCL_{10}$/Ag device recorded at different temperatures

Temperature (℃)	Set voltage (V)	Reset voltage (V)	ON state current (A)	OFF state current (A)
25	0.20	-0.20	5.61×10^{-4}	1.49×10^{-5}
30	0.20	-0.20	5.67×10^{-4}	1.48×10^{-5}
35	0.20	-0.20	5.85×10^{-4}	1.38×10^{-5}
40	0.20	-0.20	5.67×10^{-4}	1.37×10^{-5}
45	0.20	-0.20	5.70×10^{-4}	1.45×10^{-5}
50	0.20	-0.20	5.61×10^{-4}	1.49×10^{-5}
55	0.20	-0.20	5.63×10^{-4}	1.50×10^{-5}
60	0.20	-0.20	5.64×10^{-4}	1.32×10^{-5}
65	0.20	-0.20	5.61×10^{-4}	1.53×10^{-5}
70	0.20	-0.20	5.63×10^{-4}	1.39×10^{-5}
75	0.25	-0.20	5.66×10^{-4}	1.20×10^{-5}
80	0.25	-0.25	5.63×10^{-4}	1.24×10^{-5}
85	0.25	-0.20	5.62×10^{-4}	1.27×10^{-5}
Variation (%)	10.36%	6.80%	1.14%	7.80%

Supplementary Table 3. Device linewidth, crossbar size and conductance uniformity of organic memristors[8-40]

References	Linewidth (μm)	Crossbar Size	Device Variation	Conductance Uniformity	Materials System
Ref 8: ***Chem. Asian J.*** 2016, 11, 1624-1630	300	10×10	6.8%	93.2%	Al/BAzoAN/Al
Ref 9: ***ACS Appl. Mater. Interfaces*** 2013, 5, 4921-4929	200	1×5	7.0%	93.0%	Al/PIs blend/Al
Ref 10: ***Polym. Chem.*** 2013, 4, 5261-5269	400	1×6	7.4%	92.6%	Al/PVTPA-PI/Al
Ref 11: ***Nat. Commun.*** 2021, 12, 1984	0.1	8×8	8.3%	91.7%	Au/PBDTT-BQTPA/Au
Ref 12: ***J. Mater. Chem. C.*** 2018, 6, 11162-11169	25	6×6	8.5%	91.5%	Al/CMP nanofilms/rGO
Ref 13: ***J. Mater. Chem. C*** 2020, 8, 16845	200	20×20	9.0%	91.0%	Ag/PFTBDD-IrTPy/ITO
Ref 14: ***ChemElectroChem*** 2014, 1, 514-519	400	10×10	9.0%	91.0%	Al/GO–PANI/ITO
Ref 15: ***Adv. Mater. Technol.*** 2021, 6, 2000810	400	1×11	9.0%	91.0%	Cu@GaIn/PDMS-d/Cu@GaIn
Ref 16: ***Scientific Reports*** 2015, 5, 10683	1000	14×14	9.4%	90.6%	Al/PMMA:DPP-TNT/Al
Ref 17: ***Nanotechnology*** 2014, 25, 435204	150	1×6	14.5%	85.5%	Ag/PEO/Pt
Ref 18: ***J. Mater. Chem. C.*** 2017, 5, 11421-11428	600	1×3	16.6%	83.4%	Al/$(MEH\text{-}PPV)_{20}(MHPI)_{x}$-y/ITO
Ref 19: ***Adv. Electron. Mater.*** 2017, 3, 1700135	500	6×6	17.0%	83.0%	*m*-PEDOT:PSS/GO/*m*-PEDOT:PSS
Ref 20: ***Org. Electron.*** 2015, 21, 198-202	7	32×32	17.8%	82.2%	Al/PI:PCBM/Al
Ref 21: ***Chem. Commun.*** 2015, 51, 14179-14182	200	2×2	20.2%	79.8%	Al/TPA-3T-NI/Al
Ref 22: ***Chem. Commun.*** 2016, 52, 13463-13466	600	1×6	21.9%	78.1%	Ag NWs/PI (APAP)/Ag NWs
Ref 23: ***Org. Electron.*** 2018, 59, 382-388	1000	7×7	26.0%	74.0%	Al/Au NPs:lignin/Al
Ref 24: ***Appl. Phys. Lett.*** 2010, 97, 193308	3000	3×3	26.0%	74.0%	Al/LiF/P(VDF-TrFE):PEO/Au
Ref 25: ***Macromol. Mater. Eng.*** 2020, 305, 2000050	800	6×6	26.4%	73.6%	rGO/CIHP/rGO
Ref 26: ***Nature Nanotechnol.*** 2006, 1, 72-77	200	4×4	29.4%	70.6%	Al/TMV-Pt/Al

Ref 27: ***Org. Electron.*** 2021, 90, 106062	500	7×7	39.4%	60.6%	Au/PDPPBTT/ZnO/ITO
Ref 28: ***Nanotechnology*** 2008, 19, 405201	2	8×8	40.2%	59.8%	Ag/WPF-oxy-F/Ag
Ref 29: ***Polymer*** 2007, 48, 5182-5201	150	4×4	43.0%	57.0%	Al/PF6Eu/ITO
Ref 30: ***Adv. Electron. Mater.*** 2019, 5, 1800503	500	6×6	50.9%	49.1%	Al/PMMA/Au NPs/w-rGO
Ref 31: ***IEEE Trans. Electron. Dev***. 2012, 59, 3578-3582	5	8×8	56.7%	43.3%	Al/parylene-C/W
Ref 32: ***NPG Asia Mater.*** 2016, 8, e298	200	1×6	63.1%	36.9%	Al/PF_{14}-b-$Piso_n$/CNT
Ref 33: ***Nanoscale*** 2020, 12, 1484-1494	500	7×7	68.3%	31.7%	Al/Azo-Au NPs/ITO
Ref 34: ***Chin. Phys. B*** 2010, 19, 057204	0.25	16×16	/	/	Au/Ti/EA/Ti/Pt
Ref 35: ***ACS Macro Lett.*** 2013, 2, 555-560	10	3×5	/	/	Al/$PStFl2_7$-b-P_2VP_{121}/Al
Ref 36: ***IEEE Electron. Dev. Lett***. 2010, 31, 758-760	10	4×4	/	/	Al/parylene-C/W
Ref 37: ***Scientific Reports*** 2016, 6, 35273	100	10×10	/	/	Al/CuPc/ITO
Ref 38: ***Appl. Phys. Lett.*** 2007, 90, 214104	200	4×4	/	/	Al/CPMV-2QD/Al
Ref 39: ***J. Appl. Phys.*** 2006, 100, 054309	200	4×4	/	/	Al/Au nanoparticles/Al
Ref 40: ***J. Mater. Chem. C*** 2019, 7, 1491-1501	500	5×5	/	/	Al/BCPO/ITO
This work	0.05	32×32	<6.66%	>93.34%	Au/$PBFCL_{10}$/Ag

Section 7. Quantized conductance behavior of the $PBFCL_{10}$ devices

(1) Quantized conductance obtained during dc voltage sweeping

Supplementary Fig. 16a shows the current-voltage characteristics of the Au/$PBFCL_{10}$/Ag device recorded during continuous voltage sweeping in the negatively biased reset process. The stopping voltages of the continuous sweeping operation increases from -0.45 V to -0.76 V with a step of 0.01 V. By reading the device current at 0.1 V from Supplementary Fig. 16a, 32-state linear conductance quantization characteristics are demonstrated (Fig. 2d). The linearity R^2 of conductance quantization for the memristive synapses can be calculated according to the following equation,

$$R^2 = [\sum_{i=1}^{n}(\hat{y}_i - \bar{y})^2]/[\sum_{i=1}^{n}(y_i - \bar{y})^2] \quad (8)$$

where $\hat{y}_i$ and y_i represent the fitted and experimental value of device conductance, while $\bar{y}$ is the average value of the experimental device conductance. With the data shown in Fig. 2d, the linearity of conductance quantization for the present $PBFCL_{10}$ device is 0.9968. The numbers of the QC states and values of the quantized conductances of the present $PBFCL_{10}$ based

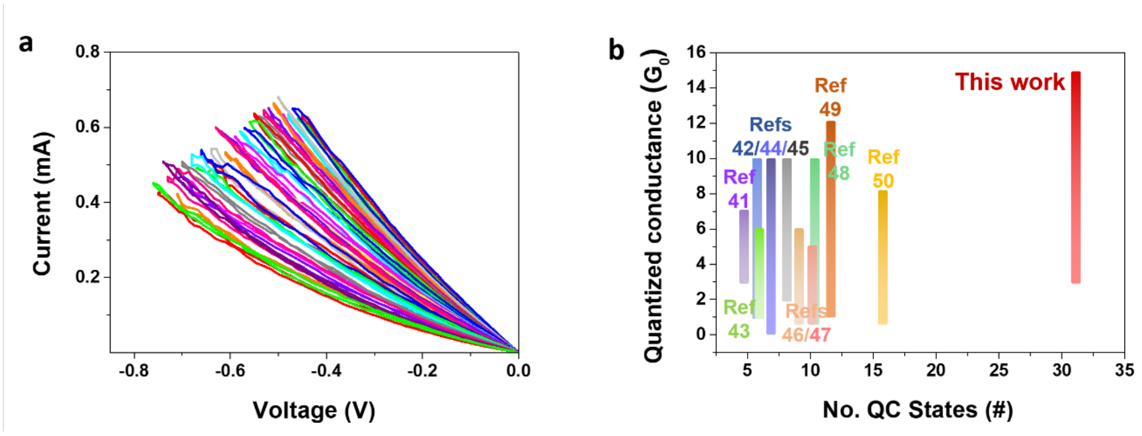

Supplementary Fig. 16| (a) Current-voltage characteristics of the Au/$PBFCL_{10}$/Ag device showing consecutive conductance modulation in the negatively biased reset process. (b) Comparison of the number of QC states and conductance distribution of the $PBFCL_{10}$ device and other silver APC based memristors as reported in Ref. 41-50.

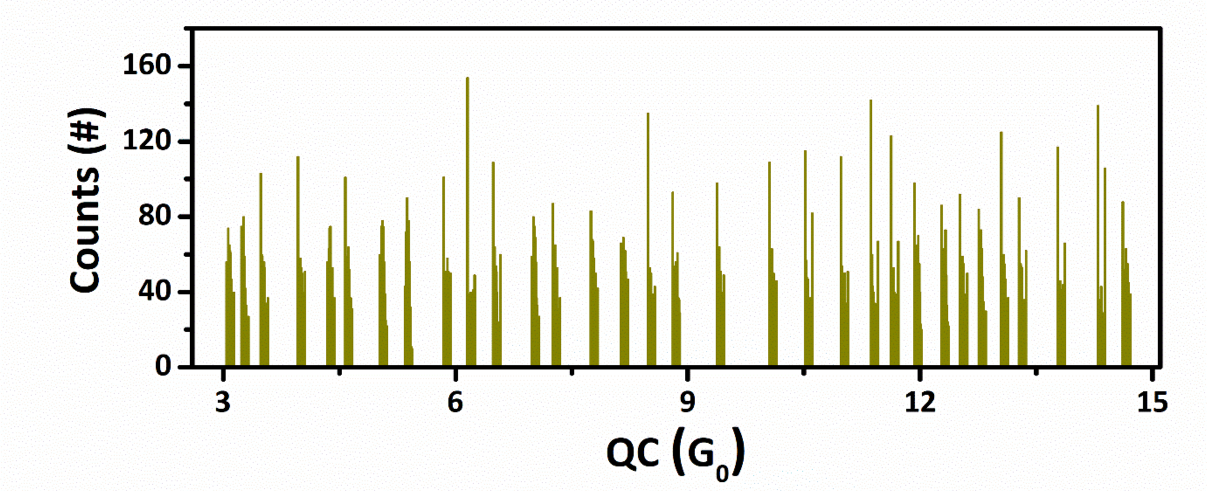

Supplementary Fig. 17 | The distribution of 32 QC states with the data retrieved from Fig. 2e.

devices, as well as those of the other silver APC based devices reported in the literatures, are summarized in Supplementary Fig. 16b[41-50]. The distribution of the 32 QC states is displayed in Supplementary Fig. 17. It is clear that these QC states do not intersect with each other.

(2) Conductance modulation in sub-QC state region

With the presence of a metallic atomic point contact in the $PBFCL_{10}$ synapse, the lowest device conductances that can be achieved during dc sweeping and pulse-mode QC modulation are 116.5 μS (~ 1.5 G_0) and 234.1 μS (~ 3.0 G_0). They are four orders of magnitude higher than that of the initial resistance state of the as-fabricated device, wherein no metal filament, atomic point contact or clusters exist in the polymer switching layer. This significant difference allows us to further decrease the device conductances and currents. In this work, we apply higher voltage pulses with the amplitudes exceeding -4.0 V to dissolute the APC, and tune the insulating gap width between the filament residue and the top electrode in the negative-feedback reset process continuously. Upon switching the device conductance to 116.5 μS though dc voltage sweeping, a voltage pulse with the amplitude of -4.0 V and width of 100 μs is applied to the

PBFCL10 device to break the Ag atomic point contact. Due to the intrinsic high resistance of the polymer switching matrix, the resultant insulating gap width between the filament residue and the top electrode decreases the device conductance from 116.5 μS to 29.5 nS abruptly (Supplementary Fig. 18). Further increasing in the voltage amplitudes from -4.0 V to -4.15 V with a ramping step of -0.01 V will continuously widen the polymer insulating gap, which in turn decreases the device conductance consecutively. As a result, a 16-step depression of device conductance from 29.5 nS to 18.6 nS, which is the initial level of the as-fabricated PBFCL10 synapse, is demonstrated. In this situation, most of the Ag clusters are annihilated and driven

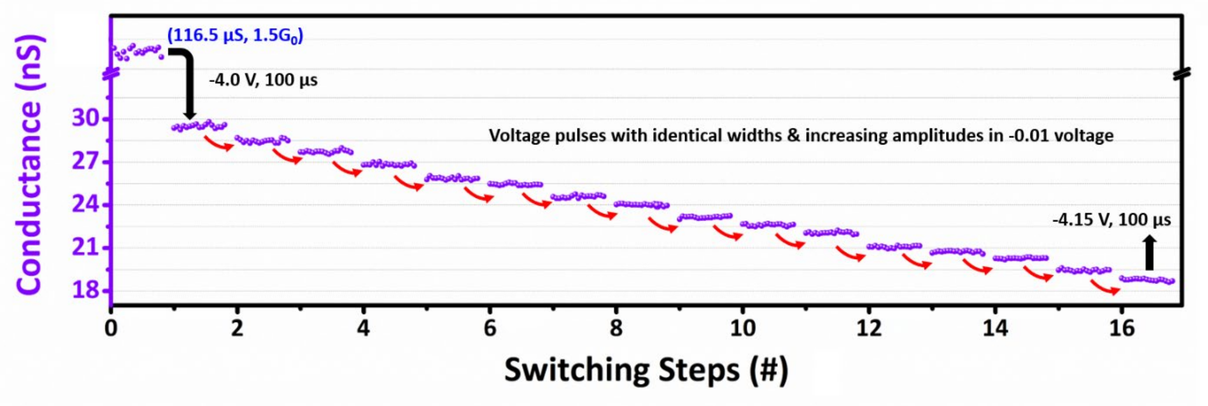

Supplementary Fig. 18| Linear stepwise of the device conductance in the sub-QC state region.

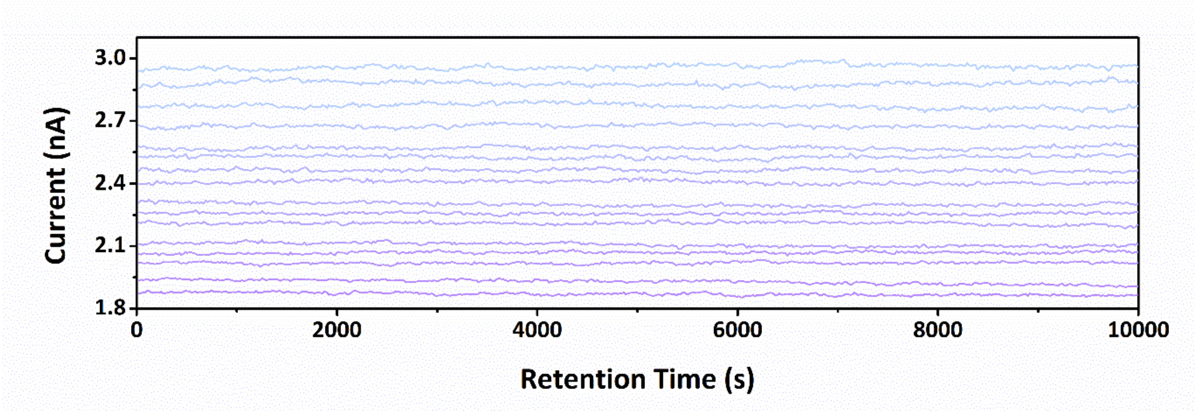

Supplementary Fig. 19| Retention performance of the 16 sub-QC states.

back to the silver electrode. The device currents and resistances during sub-QC region modulation are 2.95 nA ~ 1.86 nA (read at 0.1 V) and 3.39×10^7 Ω ~ 5.38×10^7 Ω, respectively. All 16 sub-QC states can maintain in 10^4 seconds (Supplementary Fig. 19).

(3) Reliability of conductance quantization

To evaluate the reliability of the organic synapses, we calculate both cycle-to-cycle (C2C) and D2D uniformities of the QC characteristics according to the following equation:

$$\text{Uniformity (\%)} = 1 - \left[\frac{\sum_{i=1}^{32}\left(\delta/\mu\right)_i}{32} \times 100\%\right] \tag{9}$$

where i is the sequence number of the QC states, δ is the standard deviation and μ is the average value of the device conductances in these 32 QC states. For the C2C uniformity, we use the conductances of a randomly selected device during 100 continuous switching cycles (as shown in Fig. 2f), wherein the organic synapse is switched between the 32 QC states in each cycle of operation. For the D2D uniformity, we randomly select 100 devices from the 824 devices with repeatable switching behaviors in the crossbar array and monitor their conductance evolution during switching between the 32 QC states (Fig. 2g). Note that the conductance quantization

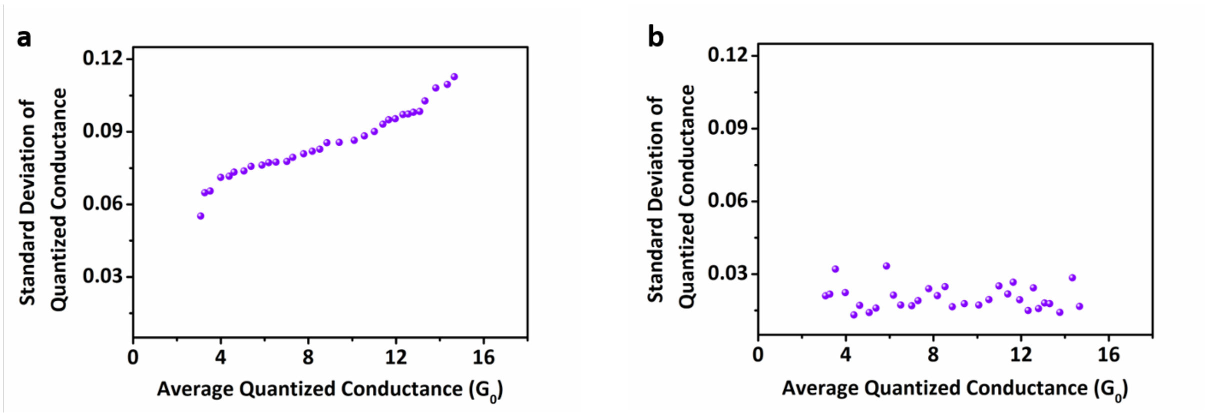

Supplementary Fig. 20| Standard deviation and average values of the device quantized conductances recorded during the (a) C2C and (b) D2D uniformity evaluations.

during both the retention and uniformity tests was programed by voltage pulses with the same width of 100 μs yet different amplitudes between -0.45 V and -0.76 V, and then read at 0.1 V. By using the device conductances plotted in Fig. 2f and 2g, as well as their standard deviation and average values summarized in Supplementary Fig. 20, the C2C and D2D uniformities of the $PBFCL_{10}$ synapses are estimated as 98.89% and 99.71%, respectively.

(4) Suppression of the cross-talking problem through 1S1R integration

In order to solve the sneak path problem of the crossbar array, an additional selector layer based on PEDOT:PSS:Ag nanoparticle composite was introduced to ensure proper selection and operation of the target synapse with the one selector-one memristor (1S1R) structure [51,52]. Due to the electron hopping between the Ag nanoparticles, threshold switching behavior is expected in the composite layer to enable the selection function. To construct the 1S1R structure crossbar array, we placed the $PBFCL_{10}$ layer formed on top of the Ag electrodes and SiO_2/Si substrate into an oxygen plasma treatment oven for 10 mins under oxygen atmosphere to make the polymer film surface hydrophilic. Afterwards, a 30 nm thick PEDOT:PSS:Ag nanoparticle layer was formed by spin-coating of 30 μL aqueous stock solution of the composite at 4000 rpm for 30 s and annealing at 100 °C in vacuum for 1 h. Finally, top electrode stripes consisting of 5 nm Ti and 40 nm Au were patterned and deposited using electron beam lithography, e-beam evaporation, and lift-off process on top of the PEDOT:PSS:Ag nanoparticle layer. 1S1R devices of Au/PEDOT:PSS:Ag nanoparticle/$PBFCL_{10}$/Ag are formed at the cross points of the orthogonally aligned top and bottom electrodes. The dimension and separation of the 1S1R devices are defined by the linewidth and separation of the top and bottom electrode strips, which are 50 nm and 85 nm, respectively. The stock solution of the PEDOT:PSS:Ag nanoparticle composite

was prepared by adding 250 μL aqueous solution of Ag nanoparticle into 500 μL aqueous solution of PEDOT:PSS. The 2 mg/mL aqueous solution of Ag nanoparticle was purchased from UIV Chem. Co. and used without further purification. The diameter of the Ag nanoparticle is 5 nm ~ 7 nm. The PEDOT:PSS solution with the concentration of 1.3wt% ~ 1.7wt% was purchased from Xi'an Polymer Light Technol. Co. and used as received.

The I-V characteristics of the Au/PEDOT:PSS:Ag nanoparticle/$PBFCL_{10}$/Ag 1S1R devices are shown in Supplementary Fig. 21a. Dc sweeping reveals that the selector layer of the 1S1R structure can be switched ON at 0.15 V. Due to the high resistance of the as-fabricated $PBFCL_{10}$ synapse layer, the device current of the 1S1R structure only increases from ~ 18.5 pA (read at 0.15 V) to 1.68 nA (read at 0.16 V). Further scanning with positively-biased voltages results in a smooth increase in the device current from nanoampere scale to sub-microampere scale, as well as an abrupt jump from 0.255 μA to 1 mA at 5.1 V, respectively. The latter corresponds the formation of a complete Ag conductive nanofilament that switch the $PBFCL_{10}$ layer to the low resistance state. Back scanning from 5.5 V to 0 V can turn the selector layer OFF at 0.1 V with the $PBFCL_{10}$ layer maintained ON, resulting in a decrease in the device current from 0.56 mA to 7.50 pA (read at 0.1 V), respectively. In the negatively biased branch, an additional series of voltage pulses with the same widths of 100 μs while increasing amplitudes from -0.45 V to -0.76 V are applied onto the 1S1R structure to modulate the conductances of the $PBFCL_{10}$ layer from the ON state to the QC state values. For example, a voltage pulse with the amplitude of -0.45 V and width of 100 μs is first applied onto the 1S1R structure. Afterwards, a dc sweeping of 0 V to -0.4 V to 0 V is used to visualize the electrical characteristics of the 1S1R structure (Supplementary Fig. 21b). As shown, the selector layer of the 1S1R structure can be turned ON

and OFF volatilely at -0.16 V and -0.10 V, respectively, while its conductance is tuned from 73.6 G_0 (read at 0.18 V in the positive branch of Supplementary Fig. 21a) to 14.6 G_0 (read at -0.18 V in the negative branch of Supplementary Fig. 21b) by the -0.45 V voltage pulse. In subsequent operations, the amplitudes of the voltage pulses increase from -0.45 V to -0.76 V with a ramping step of -0.01 V to modulate the electrical behavior of the synapse layer. Dc dual-direction sweepings between 0 V and -0.4 V indicate that the 1S1R structure shows continuously decreasing currents. By reading the device currents at -0.18 V, a 32-state linear depression of the quantized conductances from 14.6 G_0 to 3.1G_0 is obtained (Supplementary Fig. 21c). Since the device currents of the 1S1R structure exhibit ~ 7 orders of magnitude difference when the selector layer is in the volatile ON and OFF states, the sneak path problem of the QC state synapses can be solved effectively by applying appropriate voltages through the bit and word lines to selectively read the target 1S1R device in the crossbar array.

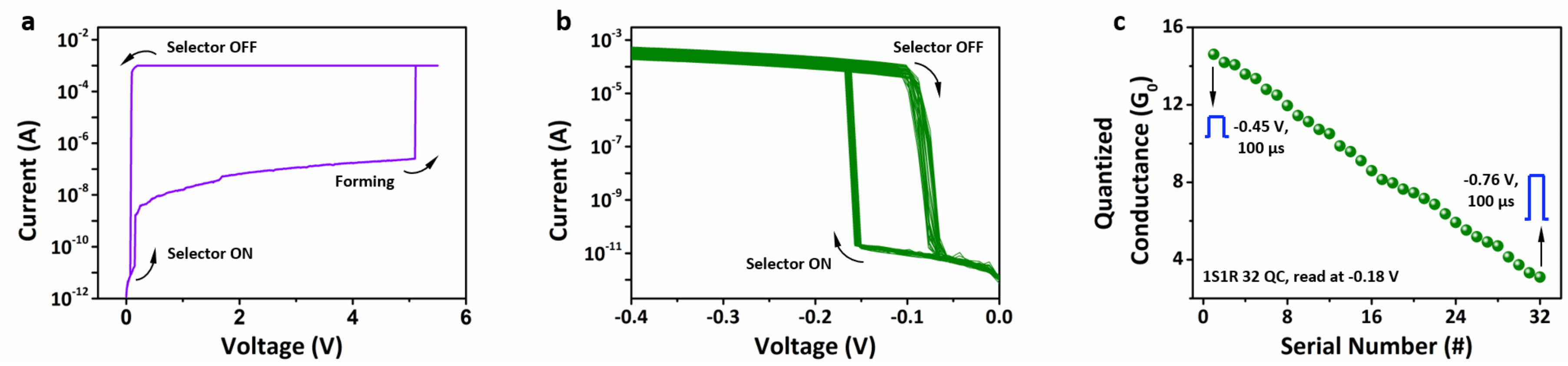

Supplementary Fig. 21│ (a) Current voltage characteristics of the Au/PEDOT:PSS:Ag nanoparticle/PBFCL$_{10}$/Ag 1S1R device showing threshold (a) bistable switching and (b) conductance quantization characteristics. (c) Evolution of the device quantized conductances in the 1S1R structure.

Section 8. Mixed-signal neuromorphic system based on the PBFCL$_{10}$ device

The circuit diagram of the PCB-FPGA controlled memristive neuromorphic system is depicted in Supplementary Fig. 22 and Fig. 3a. The home-made PCB operator is composed of a power voltage source unit, 8-bit digital-to-analog converter (DAC5578) and 12-bit analog-to-digital converter (ADC128S102) modules, an analog multiplexer (MUX508IPWR), and transimpedance amplifiers (TIAs, LTC6268). All these circuit components are controlled by the commercially available Altera Cyclone IV FPGA board. Analog voltage pulses are generated in parallel by different DACs through FPGA programming and applied as input signals to the synapse crossbar array through the row and column electrode strips. The voltage applied onto the device M_{ij} located at the ith row and j^{th} column is obtained by subtracting the jth column voltage from the ith row voltage. For example, the reference voltage V_{REFIN1} of a DAC is set as 3.3 V. Then We generate a row voltage of $V_{Ii} = \frac{240}{2^8} \times V_{REFIN1} = 3.0$ V by inputting the command bytes 11110000 into the DAC unit that controls the ith row electrode of the array. Similarly, a

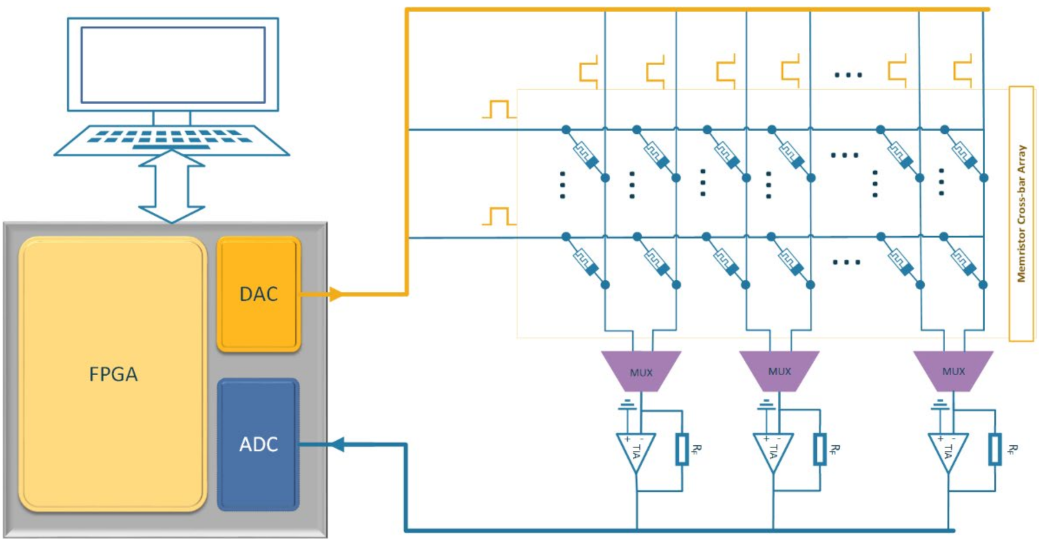

Supplementary Fig. 22│ Circuit diagram of the FPGA controlled memristive neuromorphic system.

column voltage of $V_{Ij} = \frac{46}{2^8} \times V_{REFIN1} = 0.6$ V is generated by inputting the command of 00101110 into the jth column DAC unit. As such, the exact voltage stimulus addressed onto the device M_{ij} is 2.4 V. The amplitudes of the voltage stimuli can be modified by inputting different instructions into the DAC modules, leading to either programming of the device conductance for synaptic weight updating, or execution of the vector-matrix multiplication operations through reading the current output of the crossbar array. Herein, the collection of the column current is realized through a particular MUX and further amplified by the TIA. It should be noticed that the output voltage of the TIA should not exceed the reference voltage of the ADC, and the amplification factor of the TIA can be set according to the reference voltage of the ADC. The 12-bit ADC reference voltage V_{REFIN2} is 5 V so that the transresistance value R_F of the TIA is set to 3.3 KΩ. Finally, the output voltage V_{Oj} of the TIA is converted into a digital quantity through the ADC, and further transmitted to the PC terminal for display in the digital domain. The output voltage V_{Oj} of the TIA can be expressed by $V_{Oj} = R_F \times I_{i,j} = R_F \times G_{i,j} \times (V_{Ii} - V_{Ij})$, where $I_{i,j}$ and $G_{i,j}$ are the current and conductance of the device M_{ij}. Thus, by defining the data on the PC terminal as H, the current $I_{i,j}$ and conductance $G_{i,j}$ of a particular device M_{ij} can be calculated as $I_{i,j} = \frac{V_{REFIN2} \cdot H}{2^{12} \times R_F}$ and $G_{i,j} = \frac{V_{REFIN2} \cdot H}{2^{12} \times (V_{Ii} - V_{Ij}) \times R_F}$, respectively. Updating of the synaptic weights is according to the read device conductance value and the specific algorithms of the investigated neural network, while VMM operations can be executed by simultaneously reading all the columns' current of the entire crossbar array. In addition to the updating of synaptic weights and VMM operations with appropriate voltage inputs through the DAC, MUX, TIA and ADC modules, the rest components of the neural network, including the pooling, activation, self-feedback and batch normalization, are all run in the FPGA controller.

Section 9. Synaptic plasticities of the $PBFCL_{10}$ device

In the present study, the light-weight Hopfield neural network (HNN) is employed for solving travel planning tasks, as its relatively small dimension can be completed projected onto the mixed-signal neuromorphic hardware system based on the 32×32 $PBFCL_{10}$ synapse crossbar array and the digital circuits. However, as the synaptic weight matrix of a classic HNN is maintained constant with the diagonal elements always kept zero, the network does not have any self-feedback mechanism to ensure universal convergence. The system wanders chaotically between the local minima adjacent to the initial states during iteration and the final output of the system may not be the geographically shortest solution. To overcome this issue, chaotic simulated annealing (CSA) algorithms with additional self-feedback mechanisms must be included to stabilize the system from the random walks into energy convergence during the neuron iteration (lower panel of Fig. 4a). Herein, we further study the synaptic plasticities of the $PBFCL_{10}$ synapse devices to design efficient annealing algorithm.

(1) Spike-rate-dependent plasticity

When a single voltage pulse with low magnitude (2.4 V) and a short period (10 μs) is applied onto the device, a small amount of Ag^{+} ions will be injected from the silver electrode into the $PBFCL_{10}$ thin film, get reduced back to their neutral atomic form by electrons injected from the gold cathode and accumulate into clusters near the anode interface. Since the conductive filaments that can connect both electrodes are not formed across the polymeric layer in this stage, the volatile current response of the device undergoes a quick drop followed by a gradual relaxation when the voltage stimulus is removed (Supplementary Fig. 23). The relaxation lasts for ~ 150 μs, until all the Ag atoms or clusters spontaneously diffuse back to the silver anode to lower

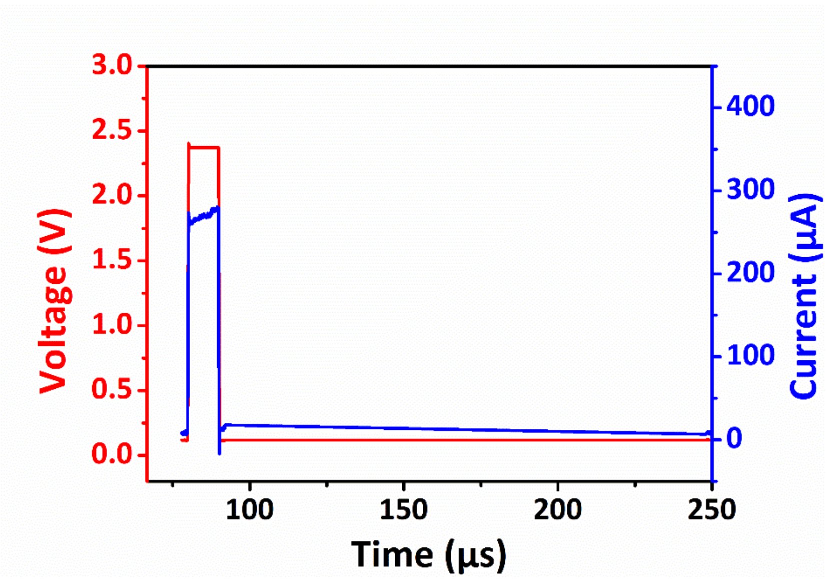

Supplementary Fig. 23| Relaxing dynamic of the Au/PBFCL$_{10}$/Ag device in response to a single voltage pulse with the amplitude of 2.4 V and width of 10 μs.

the total energy of the neuromorphic system. When a dozen of the non-overlapped pulses with the same magnitude, width, yet a time interval (160 μs) longer than the relaxation period is applied, the lack of interaction between the individual pulse's influences on the device results in almost unchanged current responses (Supplementary Fig. 24a). As the spiking interval decreases to shorter than the relaxation period, the Ag atoms injected into the polymer matrix do not have enough time to relax back to their original state. Interaction of the subsequent pulses will promote the migration of the metal ion further towards the cathode and lead to an obvious increase in the device's currents (Supplementary Fig. 24b to 24f). Such spiking-rate-dependent plasticity (SRDP) of the PBFCL$_{10}$ based synapse, which obeys the mathematical expression:

$$\Delta\omega = C_1 \exp(-t/\tau_1) + C_2 \exp(-t/\tau_2) \quad (10)$$

where $\Delta\omega$ is the change of device conductance, C_1 and C_2 are constants of 3000 and 95, t is the time interval between neighboring pulses, τ_1 and τ_2 are time constant of the SRDP curves and derived to be to 4 μs and 43 μs, respectively, is plotted in Supplementary Fig. 25a. Subsequently,

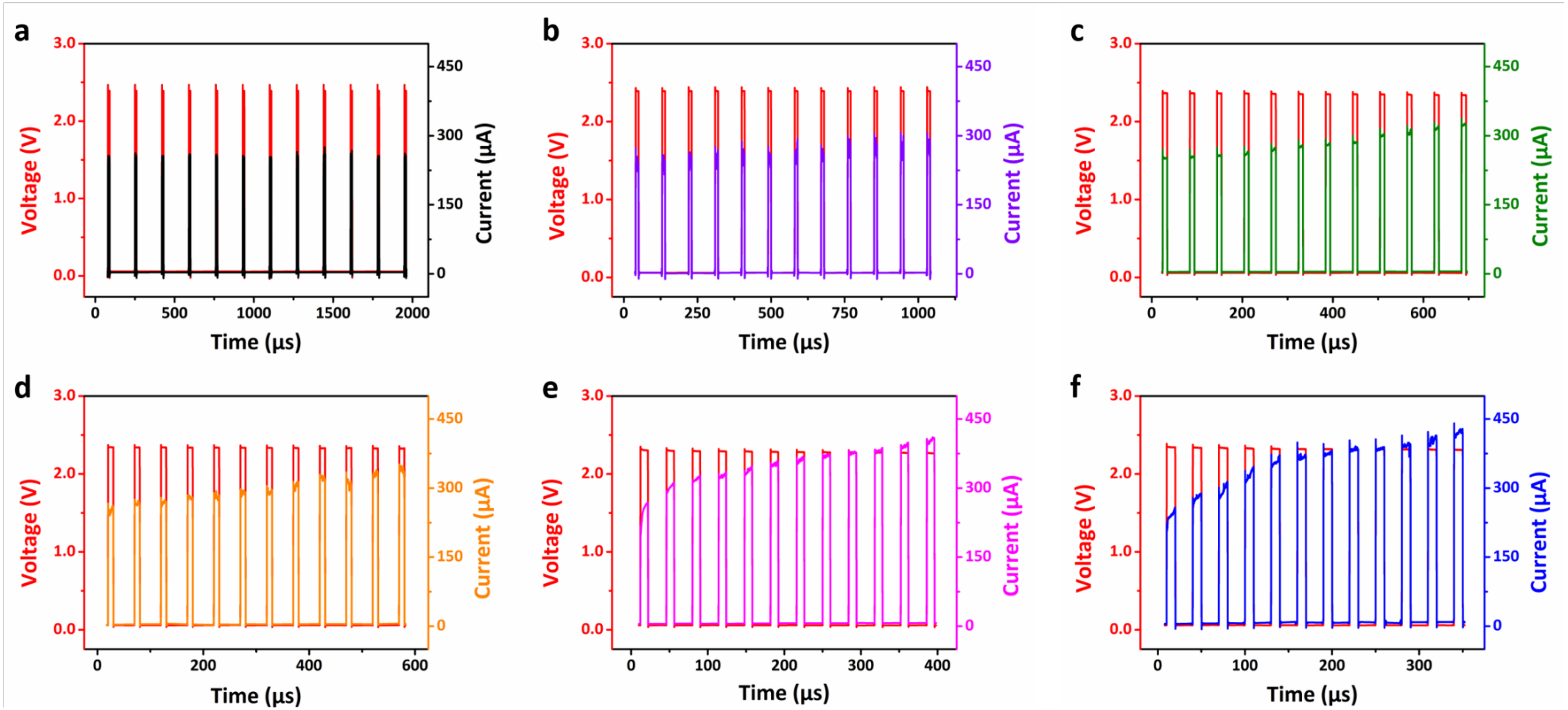

Supplementary Fig. 24│ Transient currents of the Au/PBFCL$_{10}$/Ag device in response to voltage pulse trains with the same pulse numbers of 12, amplitude of 2.4 V, width of 10 μs while different intervals of (a) 160 μs, (b) 80 μs, (c) 50 μs, (d) 40 μs, (e) 24 μs and (f) 20 μs, respectively.

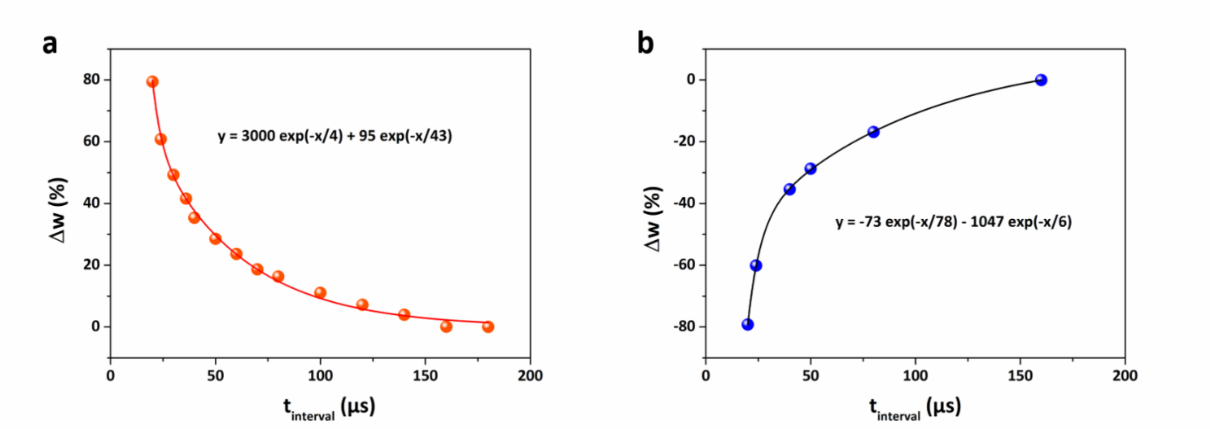

Supplementary Fig. 25 | Spike-rate-dependent plasticity (SRDP) of the $PBFCL_{10}$ device showing conductance variation with the increase of voltage pulse interval time in both the (a) potentiation and (b) depression processes.

SRDP characteristics was also recorded in the depression branch of the present $PBFCL_{10}$ devices, under the stimulation of another sets of voltage pulse trains with the amplitudes of -2.4 V and intervals of 160 μs to 20 μs (Supplementary Fig. 25b and 26), respectively. Similar SRDP characteristics during the weight potentiation and depression procedures were observed in both biological and other memristive synapses[53-55].

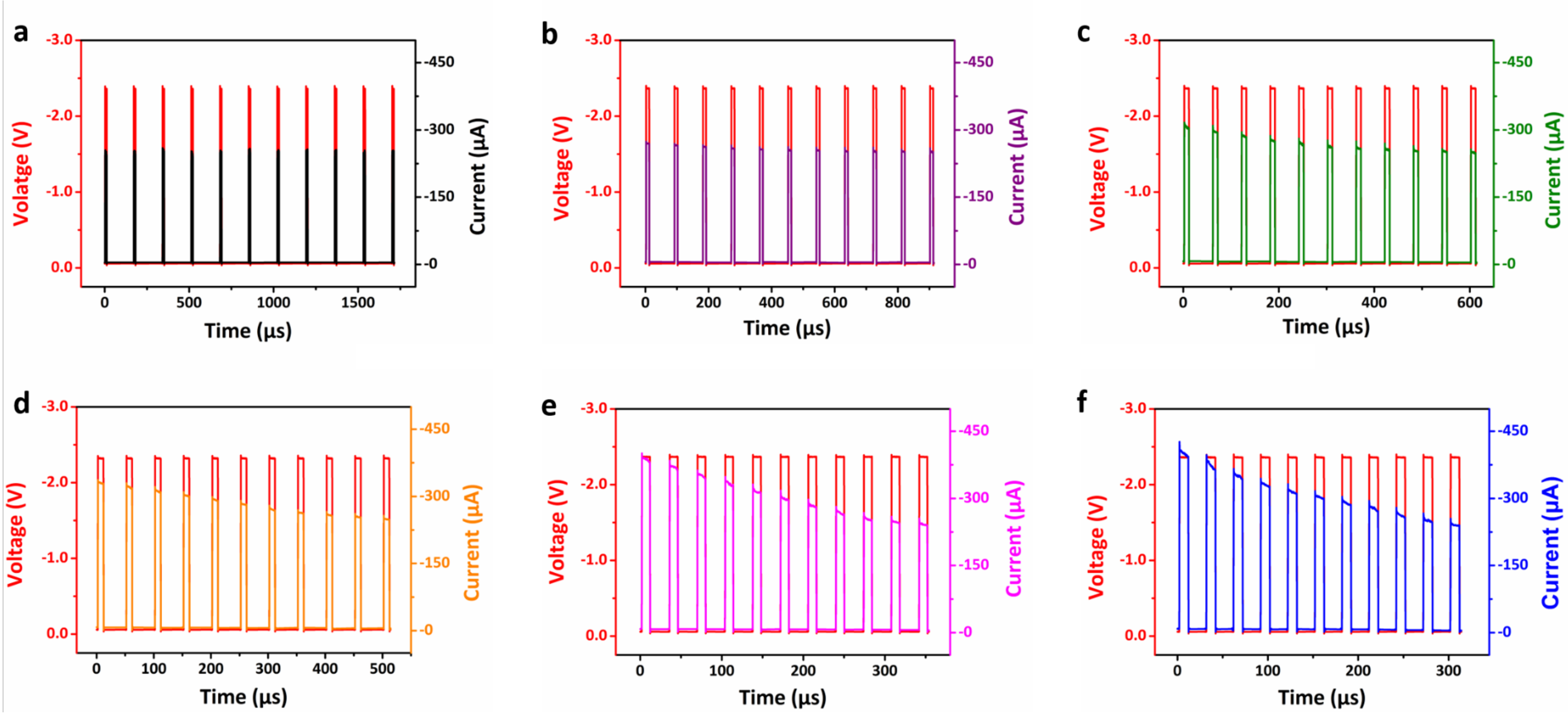

Supplementary Fig. 26. The transient currents of the Au/PBFCL$_{10}$/Ag device in response to the depression process with the same pulse numbers of 11, amplitude of -2.4 V, width of 10 μs while different intervals of (a) 160 μs, (b) 80 μs, (c) 60 μs, (d) 40 μs, (e) 24 μs and (f) 20 μs, respectively.

(2) Spike-timing-dependent plasticity

The spiking-time-dependent plasticity (STDP) of the $PBFCL_{10}$ device is explored with the Ag electrode acting as the pre-synaptic neuron and Au electrode acting as the post-synaptic neuron, respectively. The interplay between the pre- and post-synaptic spikes with various timings can elaborately control the dynamic migration behavior of silver atoms/clusters in the polymer layer, which gives rise to STDP responses in the first and third quadrants of Supplementary Fig. 27. The relationship for the $\Delta\omega$ and timing difference $\Delta t_{Post\text{-}Pre}$ can be fitted as below:

$$\Delta\omega(t_{Pre}, t_{Post}) = A\exp(-\Delta t_{Post\text{-}Pre}/\tau) \quad (11)$$

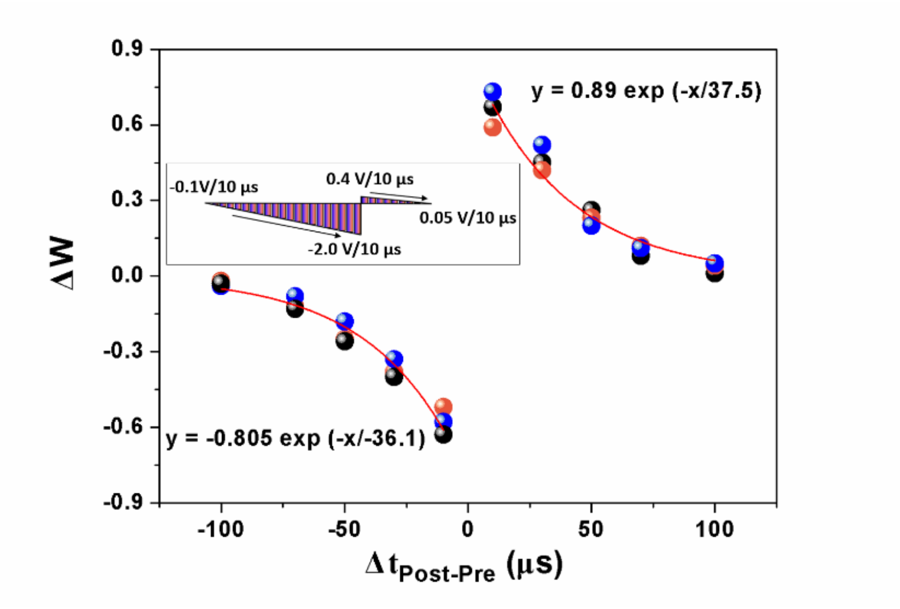

Supplementary Fig. 27| Spike-timing-dependent plasticity (STDP) of the $PBFCL_{10}$ device showing synaptic potentiation and depression in the positive and negative domain of the time interval between the pre-synaptic and post-synaptic spikes, respectively. Increasing (decreasing) the time interval between the spikes will result in a low (high) potentiation or depression effect, respectively. Inset shows the waveshape of the pre- and post-synaptic spikes used in the spiking-timing-dependent plasticity measurements. Each spike is composed a train of 20 negatively-biased voltages with the width of 10 μs and amplitudes increasing from -0.1 V to -2.0 V in a ramping step of -0.1 V, and a train of 8 positively-biased voltages with the width of 10 μs and amplitudes decreasing from 0.4 V to 0.05 V in a declining step of 0.05 V, respectively.

where A is a constant of 0.89 and -0.805 for first and third quadrants, respectively, $\Delta t_{Post\text{-}Pre}$ represents the time interval between pre- and post-synaptic spikes and τ is a time constant of 37.5 and -36.1 for potentiation and depression, respectively.

Section 10. Circuit design of the STDP comparator

Hardware implementation of the STDP comparator circuit was designed by Verilog coding, complied on Altera Quartus ii platform and physically realized with the commercial Altera Cyclone IV FPGA board. Supplementary Fig. 28 displays the register transfer level (RTL) schematic diagram of the STDP comparator generated by the Verilog coding. In the STDP comparator circuit, the PLL is a phase-locked loop that acts to produce a frequency division clock of 0.01 Mhz and 0.1 MHz, provided to the counter latch module and the pulse generation module, respectively. The cnt_latch is a count latch module with an input clock frequency of 0.01 Mhz. It converts the travel times of the routes predicted by the network (T_2) and the initial set value T_1 into voltage signals. When the counting function of the cnt_latch module completes, a high-level voltage signal will be transmitted from its out port and activates the wave_creat pulse generation module to generate a 14-bit parallel digital signal (DAC_DATA1, DAC_DATA2). The 14-bit parallel digital signal, together with the clock signal (DACA_CLK, DACB_CLK) and data write signal (DACA_WRT, DACB_WRT), will be transmitted to the DAC module and converted into an analog voltage signal to generate the STDP pulses shown in the inset of Supplementary Fig. 27. The STDP pulses are then applied to the two electrodes of the synapse. By monitoring the incremental/decremental change of the device's output current, the relative magnitudes of T_1 and T_2 can be obtained through the STDP characteristics of the device. An increase (decrease) in the output current of the as-designed comparator circuit indicates a smaller (larger) T_2 than T_1. Supplementary Fig. 29 demonstrates the wave-shapes of the STDP pulses and experimental outputs of the $PBFCL_{10}$ synapse device in the comparator circuit. We applied a current of 50 μA to the synapse through the current source and observed the corresponding voltage

output change of the device. As shown in Supplementary Fig. 29a, when T_1=5 and T_2=6, that is, the input count of cnt_latch_pre is 5, and the input count of cnt_latch_post is 6, the corresponding pre-pulse V_{pre} is applied to the synapse before the post-pulse V_{post}. At a result, the device conductance increases and the voltage V_M decreases accordingly. When T_1=6, T_2=5 (Supplementary Fig. 29b), that is, the input count of cnt_latch_pre is 6, and the input count of cnt_latch_post is 5, corresponding to the pre-pulse V_{post} applied to the synapse later than the post-pulse V_{post}. Consequently, the device conductance decreases and the voltage V_M increases accordingly. These results confirm that the organic synapse based STDP comparator circuit functions normally as expected.

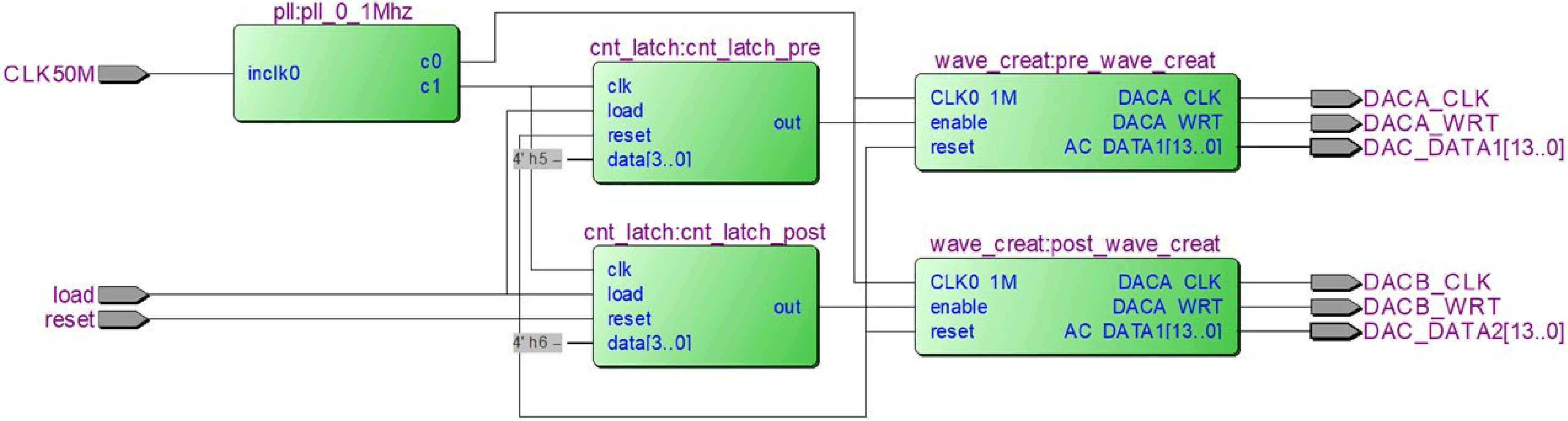

Supplementary Fig. 28 | Register transfer level (RTL) schematic diagram of the STDP comparator circuit generated by Verilog coding.

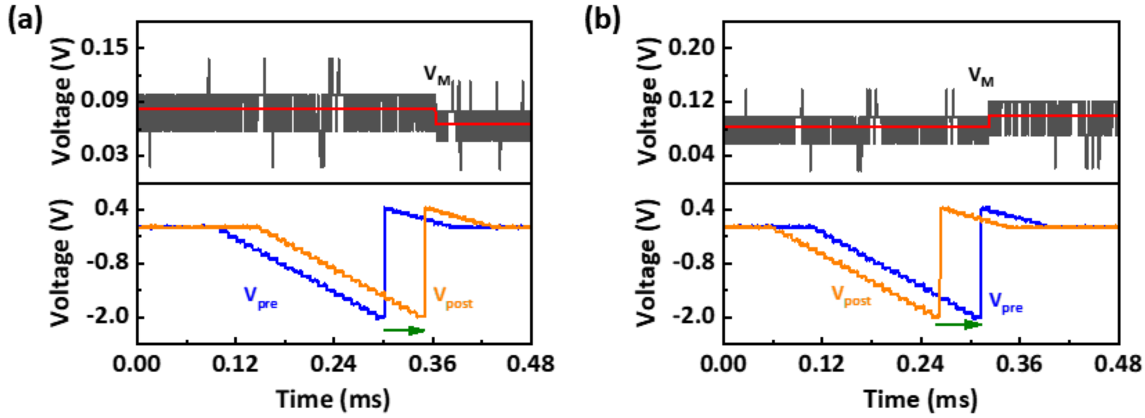

Supplementary Fig. 29│ The wave-shapes of the STDP pulses and experimental outputs of the $PBFCL_{10}$ device in the comparator circuit for synaptic potentiation (a) and depression (b), respectively.

Section 11. Operation of the bioinspired Hopfield neural network

(1) Sketching of the Minhang District map

For travel route planning in real-world applications, both the geographical distance between the departure and destination sites, and the travelling speed (associated with mode of transport and traffic conditions) should be considered to find a path with relatively shorter distance and time. For simplicity, we sketch a map on which 32 locations of interests are involved and arranged in an ordered manner along the longitudes and latitudes (Supplementary Fig. 30). This map is a simplified version of Minhang District, Shanghai Municipality. All the involved locations are labeled by Arabic numerals and map coordinates from the bottom-left corner to the up-right corner of the map. Among them, only parts of the locations are directly interconnected with each other while the others are not. According to the travelling speed that each inter-location path can accommodate, these paths are categorized into three traffic circumstances with the relative travelling speeds of 5, 3 and 2 in green, blue and red, respectively (Supplementary Fig. 33a and 33d).

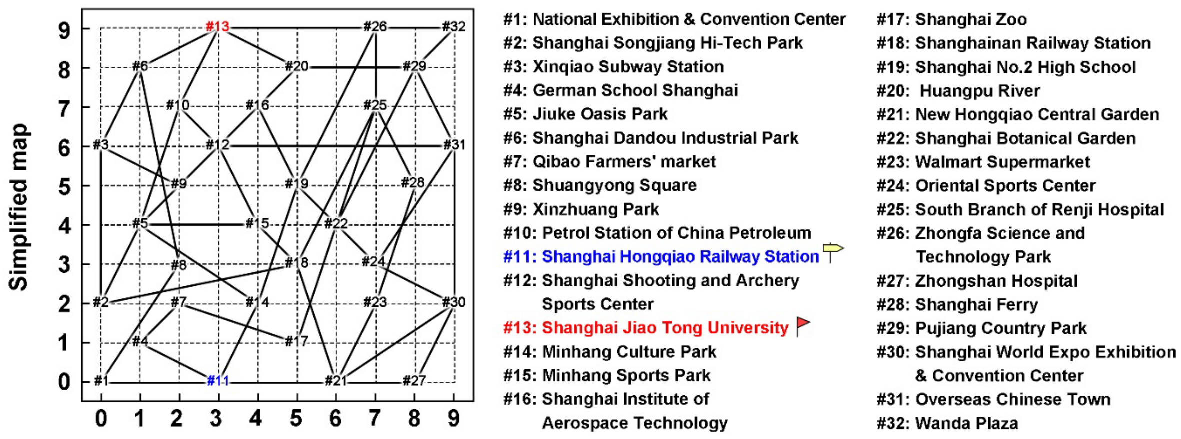

Supplementary Fig. 30│ A simplified local map of the Minhang District of Shanghai City used for travel path planning in the present study. Each of the locations is labeled with its real-world name and numbered with an Arabic numeral and a pair of geographical coordinates from the bottom-left corner to the up-right corner of the map, respectively.

(2) Method of network iteration

The operation of a Hopfield neural network (Supplementary Fig. 31) can be mathematically expressed as following[56]:

$$x_i(t) = \frac{1}{1+e^{-\frac{y_i}{\varepsilon}}} \tag{12}$$

$$y_i(t+1) = ky_i(t) + \alpha\left(\sum_{j=1}^{n} w_{ij}x_j(t) + I_i\right) - z_i(t)(x_i(t) - I_0) \tag{13}$$

$$z_i(t+1) = (1-\beta)z_i(t) \tag{14}$$

where x_i, y_i, z_i represent the output, internal state variable and self-feedback synaptic weight of the neuron i, respectively. In this study, the synaptic weights of the HNN are represented by the conductances of the $PBFCL_{10}$ synapse, according to the Supplementary Table 4. Among the above expressions, Equation 12 is the activation function of the network with x_i being a sigmoid function of y_i and ε as a steepness parameter. Equation 13 describes the iterative updating of the internal state of the neuron i. The first term is the internal state of the neuron i at time t with a damping factor k ($0 \leq k \leq 1$). The second term represents the influence of all other neurons as well as external stimulus I_i on the neuron i at time t, which has been scaled by a positive constant α. w_{ij} is the normalized connection weight from neuron j to neuron i and represents the direct distance between locations i and j. The distances between all the unconnected locations are set as the square-foot of 200, which are the farthest distance along the diagonal of the map. They are normalized as $w_{ij} = 1$ in the weight matrix. Classic Hopfield neural network only uses the first two terms for neuron state updating while the as-obtained optimal state may not be the global energy-minimum state necessarily. In order to force the system to converge into the global optimum, a third term of the neuron's self-feedback is included to introduce chaotic dynamics into the system. Herein, I_0 is a constant, while z_i ($z_i \geq 0$) describes the chaotic degree

of the neuron. Note that the weight matrix will be maintained constant during neuron state updating, except for the decreasing diagonal self-feedback weights along with the iteration. When chaos with fixed magnitudes is used, z_i is a constant and I_0 equals to 0. The system can transit from the present local minimum into another energy state easily. However, the inclusion of fixed chaos may also stimulate an undesirable escaping of the system from the global optimum into a local minimum state with a relatively higher energy level. The network keeps iteration until the pre-defined numbers of epochs have been executed completely. To ensure an accurate convergence of the system, a chaotic simulated annealing algorithm (CSA) with time-dependent z_i and non-zero constant I_0 should be incorporated. Generally, $z_i(t)$ is endowed with a random value during network initialization while the damping factor β controls the annealing (decreasing) rate through Equation 14. The main purpose of involving I_0 is to guarantee the direction of convergence towards gradually lowering energy. Therefore, the selection of a proper annealing algorithm is the key to achieving fast and accurate convergence of the network into a global energy-minimum state (see the lower panel of Fig. 4a).

The implementation of the above-mentioned CSA annealing algorithm is operated on regulating the diagonal self-feedback weights of the network, which has been mapped onto the conductance modulation of the 32×32 crossbar array of $PBFCL_{10}$ synapse array through a linear transformation $G_{ij} = aw_{ij} + b$. In total 17 conductance values have been used to update the self-feedback weights in this study. Upon each iteration step, the network will generate a potential route and check its validity with Equation 15,

$$E_1 = \frac{U_1}{2}\left\{\sum_{i=1}^{m}\left(\sum_{j=1}^{m} x_{ij} - 1\right)^2 + \sum_{j=1}^{m}\left(\sum_{i=1}^{m} x_{ij} - 1\right)^2\right\} \quad (15)$$

where U_1 is a positive coupling constant, x_{ij} is the neuron output that describes whether the

Supplementary Table 4. Representation of synaptic weights by device conductances

No.	Synaptic Weight	Device Conductance (μS)
**1**	1.00E-05	1134.83
**2**	0.005	1111.65
**3**	0.0288	1066.36
**4**	0.0526	1034.45
**5**	0.0764	1016.72
**6**	0.100	991.18
**7**	0.111	975.93
**8**	0.157	954.63
**9**	0.176	926.23
**10**	0.236	906.03
**11**	0.248	890.36
**12**	0.283	849.84
**13**	0.314	813.73
**14**	0.351	778.71
**15**	0.400	728.18
**16**	0.471	682.55
**17**	1	119.53

direct visiting path from location i to location j is involved in the designed route, and E_1 is the restriction part of the system energy in the current moment. In case that the current route is valid, each halfway location involved in the designed route will be visited once, and only one halfway location will be visited at one time. This corresponds to the output matrix in which only one neuron in each column or row is activated with its output approaching 1. All the other neurons in the specific column or row are inactivated and thus output ~ 0. As such, the pseudo zero-energy state E_1 of the system serves as the validity criterion of the planned route.

For the valid route obtained with neuron state iteration, the traveling time between the departure point and destination will be calculated with the following Equation 16:

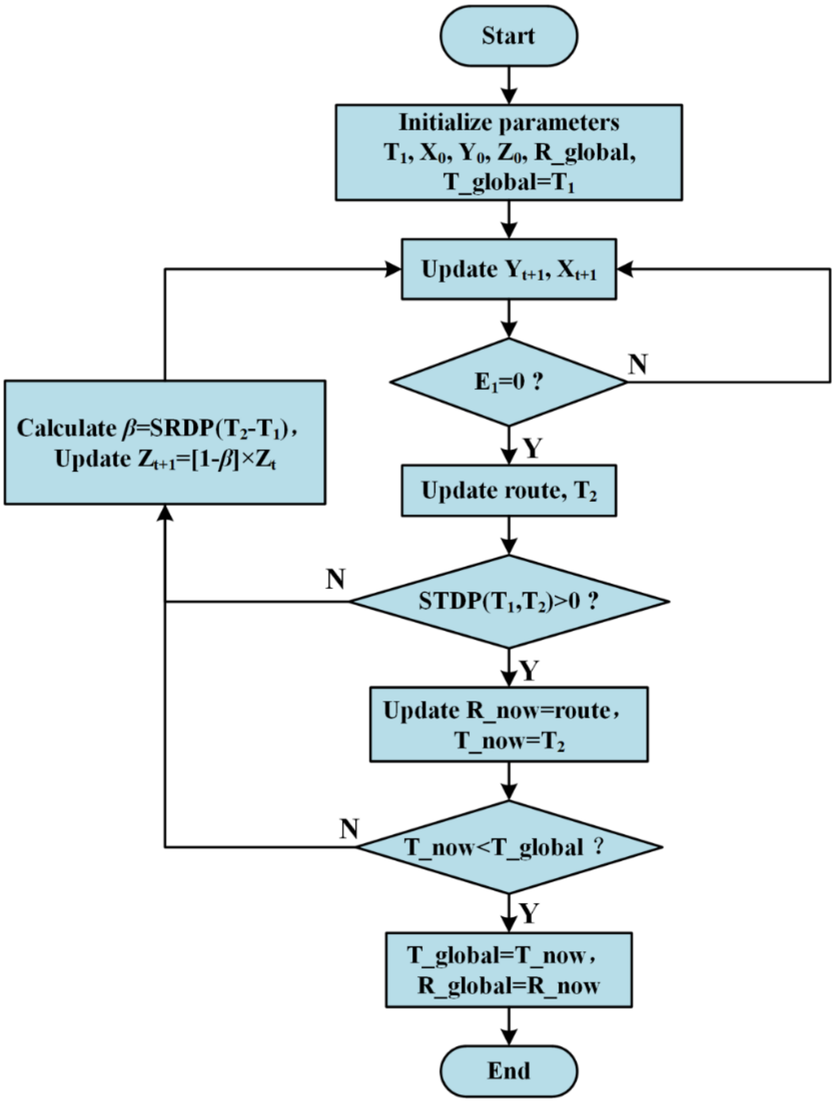

Supplementary Fig. 31 | Flow chart of the bioinspired Hopfield neural network enhanced with SRDP based chaotic stimulated annealing (CSA) algorithm.

$$T_2 = \sum_{i=1}^{m}\sum_{j=1}^{m}\sum_{k=1}^{m}\left(x_{k,j+1} + x_{k,j-1}\right)x_{ij}\frac{d_{ik}}{v_{ik}} \tag{16}$$

where d_{ik} and v_{ik} are the interconnected distance and pre-determined travelling speed between the locations i and k, respectively. If T_2 is shorter than T_1, it will be used to replace T_1 as a temporary optimal solution. The corresponding energy of the present route can be obtained by scaling T_2 with an additional coupling parameter U_2:

$$E_2 = \frac{U_2}{2}T_2 \tag{17}$$

The total energy of the system is obtained by the addition of E_1 and E_2. Afterwards, the damping factor β will be retained for subsequent neuron state updating. If T_2 is longer than T_1, the present route is not desirable in either the energy domain or the real-world travel cases. In such a case, T_1 will be retained while ΔT corresponding to $|T_1-T_2|$ is taken into Equation 18 to recalculate β with the two-exponential SRDP dynamics of the $PBFCL_{10}$ synapse device to shift the system to another domain with potentially lower energy:

$$\beta(\Delta T) = 3000e^{-\frac{\Delta T}{4}} + 95e^{-\frac{\Delta T}{43}} \quad (18)$$

where 3000, 95, 4 and 43 are constants derived from Equation 10 and Supplementary Fig. 25. It is noteworthy that β mentioned herein is a function of the travelling time, with which the annealing process will become self-adaptive. Thus, the prediction rate and accuracy of the Hopfield neural network convergence will be enhanced through bioinspired spiking plasticities.

In the present study, we set the same parameters of k=0.9, ε=0.004, W_1=W_2=1 and I_0=0.5 for all the linear, exponential and SRDP-based annealing algorithms during network initialization. The initial self-feedback synaptic weight was set as z_0=0.1, while the internal states of all the neurons were randomly initialized within (-0.1, 0.1). For linear annealing, the self-feedback expression is $z(t+1)=z(t)-\beta$, with parameter β being used to control the annealing speed. The expression of the exponential and bioinspired annealing is given in Supplementary Equation 14, with fixed β of 0.004, 0.008 and 0.012, as well as varying β according to the SRDP characteristics (Supplementary Equation 18), to control the annealing speed.

(2) Results of travel route planning

Initially, the outputs of the neurons are endowed randomly between 0.46 and 0.54, while

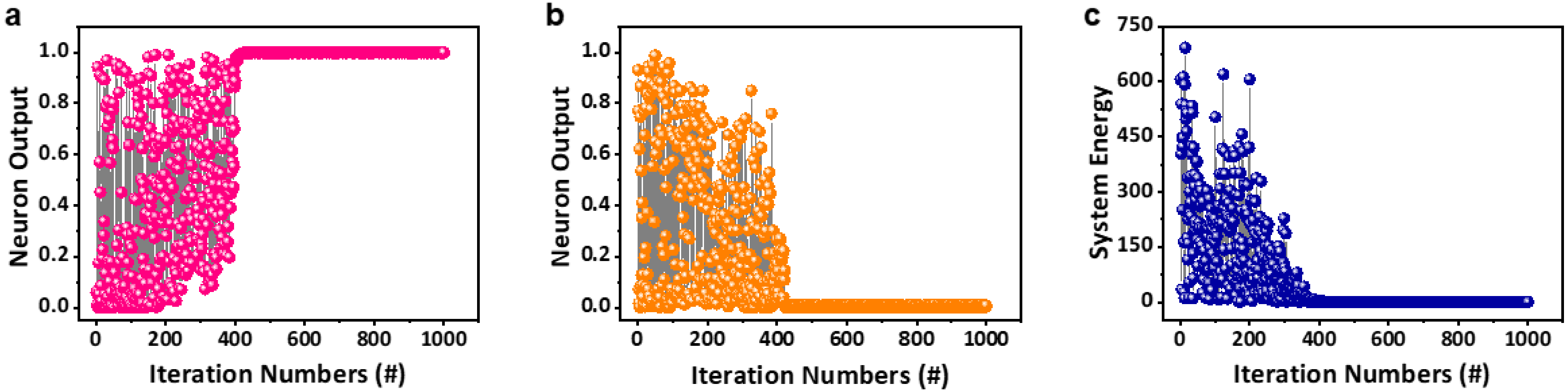

Supplementary Fig. 32 | Evolution of (a) an activated neuron's output x, (b) an inactivated neuron's output x and (c) system energy during 1000 iterations, respectively.

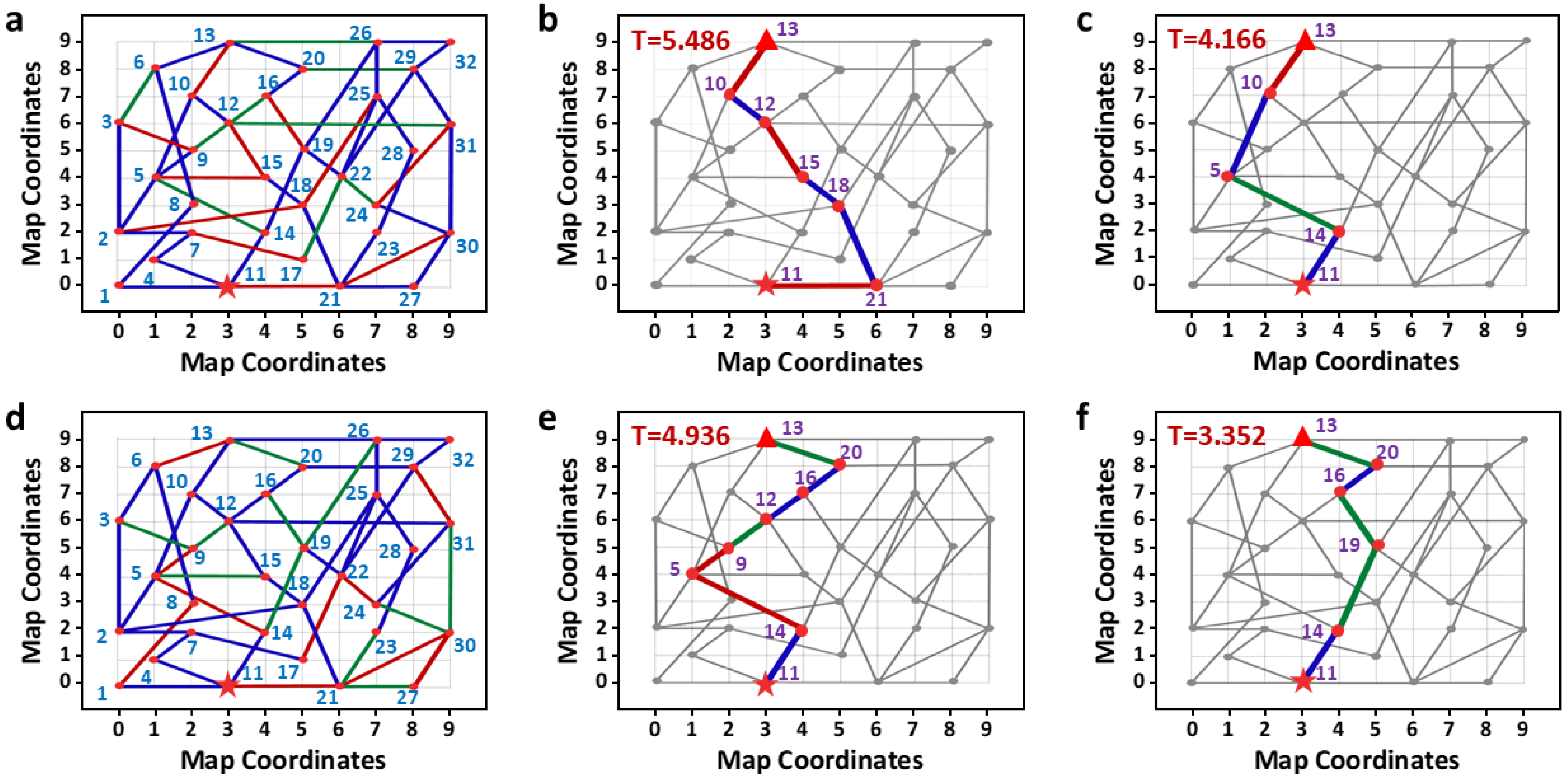

Supplementary Fig. 33 | **a,d** The simplified local map of Shanghai Municipality used in this study with speed plan of V1 and V2, respectively. 32 locations are involved and defined with their respective geographic coordinates in the map. The star and triangle symbols label the departure and destination points. The inter-location paths represented by green, red and blue colors have different relative travelling speed of 5, 3, and 2, respectively. **b,e** The valid route obtained by the BHNN upon one step of neuron state updating. **c,f** The optimized route with shortest travelling speed as predicted by the BHNN upon 1000 cycles of neuron state updating.

the conductance matrix of the 1024 $PBFCL_{10}$ synapses in the 32×32 crossbar array is programmed according to the synaptic weight initializing scheme of the network (Fig. 3d). During iteration, the synaptic weight matrix of the bioinspired HNN is maintained constant during neuron updating, except for the diagonal self-feedback weights that decrease *in-operando*. In the meanwhile, the outputs of the activated neurons increase, while the outputs of the inactivated neuron and the total energy of the network decrease (Supplementary Fig. 32). The abscissa in the output matrix describes the visiting sequence of locations (Fig. 4c), and the ordinate ascending is the labeling number of the 32 locations. The strength of each pixel represents the activation level of the neuron and thus possibility of visiting the respective location during the specific visiting step. As shown in Supplementary Fig. 33a and 33b, a valid route (11-21-18-15-12-10-13) has been obtained after one step of neuron state updating, with a travelling time of 5.486. As the iteration continues, the outputs of the activated neurons increase and finally approach 1 (Supplementary Fig. 32a), whereas both the outputs of the inactivated neurons and the total energy of the network decreases (Supplementary Fig. 32b and 32c). With the iteration reaching 200 epochs, the neuron state will convergence to local optima, and the pixel strength evolves to the range of 0.07 to 0.83 (middle panel of Fig. 4c). Note that the synapses 11 and 13 that correspond to the labeling numbers of the departure (Hongqiao Railway Station) and the destination (Shanghai Jiao Tong University) points are now activated in the twenty-eighth and thirty-second columns of the output matrix, the predicted route of 11-21-18-15-12-10-13 can therefore be depicted by connecting all the activated neurons between these columns sequentially. As shown in Fig. 3e, the conductance matrix *in-operando* updated by the 32×32 $PBFCL_{10}$ synapse crossbar array only deviates by 0.23% from the expected values predicted according to the

synaptic weight updating scheme of the BHNN, Supplementary Table 4 and the following equation,

$$\text{Deviation} = \left[\frac{\sum_{i=1}^{1024}\left[{(G_E - G_T)}/{G_T}\right]_i}{1024} \times 100\%\right] \tag{19}$$

where G_E is the experimentally updated conductance of the ith $PBFCL_{10}$ device in the 32×32 crossbar array of the neuromorphic hardware system, while G_T is the theoretical value predicted according to the synaptic weight updating scheme of BHNN and Supplementary Table 4, respectively. The small deviations in Fig. 3e confirm that device conductance and synaptic weight updating can be accurately achieved in the present organic synapse based hardware system, which therefore is reliable for executing practical neuromorphic computing tasks. Upon iterating for more than 420 epochs, a 100% activated neuron appears in each column of the output matrix, with which the desired route of 11-14-5-10-13 as shown in the right panel of Fig. 4c can be obtained similarly. Again, the final conductance matrix updated experimentally on the $PBFCL_{10}$ synapse crossbar array is also in close proximity to the theoretical values (Fig. 3f). Additionally, the traffic congestion circumstances of the same route may not be identical during the day, which will lead to different travel plans between the starting and destination locations. As demonstrated in Supplementary Fig. 33d-33f, iteration with speed plan V2 will generate a different route between the railway station and the university.

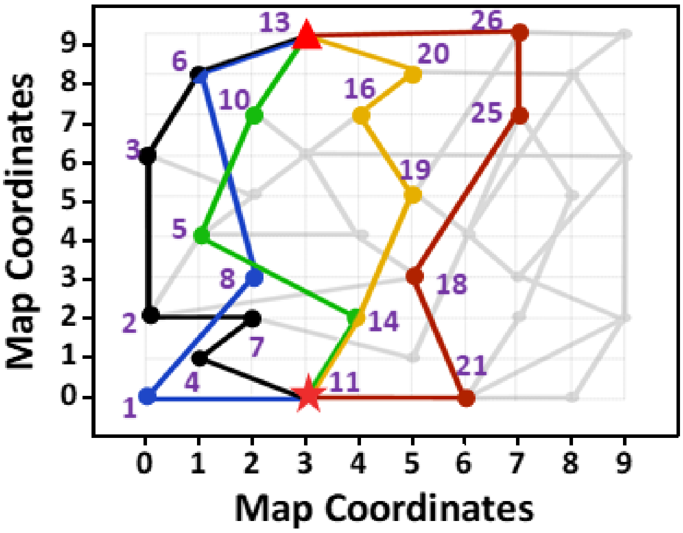

Supplementary Fig. 34| Comparation of planed routes predicted by network initialization (blue line), classic HNN (black line), linear annealing enhanced HNN (red line), and bioinspired HNN with iteration of 200 (yellow) and 420 (green), respectively.

(3) Influence of damping factor *β* on the network iteration efficiency

To evaluate the iteration efficiency of the bioinspired Hopfield neural network, we further explore the evolution of the output matrix for the activated neurons with different fixed damping factor β. As plotted in Supplementary Fig. 35, a smaller β value corresponds to a relatively slower annealing rate. The iteration cycles needed by the system to reach global optimum decrease from 765, 615 to 575 for fixed β of 0.004, 0.008 and 0.012, respectively. Nevertheless, a smaller β value will on the other hand lead to a better convergence of the network, giving rise to the activated neuron's final outputs of 0.989±0.006, 0.938±0.030 and 0.927±0.030 for the fixed β of 0.004, 0.008 and 0.012, respectively. In comparison, the SRDP based CSA algorithm shows greatly reduced attenuation factor β ranging from 1.46 e^{-2} to 3.37e^{-7}, but demonstrates a fast (420 epochs) yet more accurate convergence (final output of the activated neuron, 0.999 ± 0.001) to the global optimal state (Supplementary Fig. 32a and 32c).

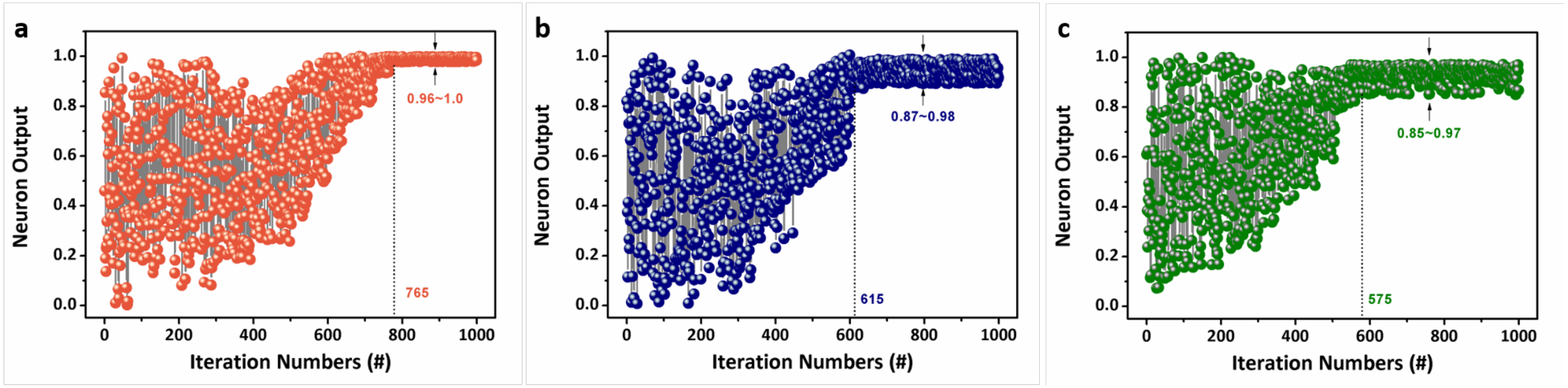

Supplementary Fig. 35│ Output x evolution of the activated neuro during exponential annealing with fixed β of (a) 0.004, (b) 0.008 and (c) 0.012, respectively.

References

1. Soccio, M., Martinez-Tong, D. E., Alegria, A., Munari, A. & Lotti, N. Molecular dynamics of fully biobased poly(butylene 2,5-furanoate) as revealed by broadband dielectric spectroscopy. *Polymer* **128**, 24-30 (2017).

2. Hu, H., Zhang R., Zhu, J. et al. Biodegradable elastomer from 2,5-furandicarboxylic acid and ε-carprolactone: effect of crystallization on elasticity. *ACS Sustainable Chem. Eng.* **7**, 17778-17788 (2019).

3. Milano, G. et al. Quantum Conductance in Memristive Devices: Fundamentals, Developments, and Applications. Adv. Mater. 34, 2201248 (2022).

4. Terabe. K. et al. Quantized conductance atomic switch. Nature. 433, 47-50 (2005).

5. Brandbyge, M. et al. Quantized conductance in atom-sized wires between two metals. 52, 8499-8514 (1995).

6. Jayasekharan, T. & Sahoo, N. K. Characterization of [peptide+$(Ag)_n]^+$ complexes using matrix-assisted laser desorption/ionization time-of-flight mass spectrometry. *Rapid Commun. Mass Spectrom.* **24**, 3562-3566 (2010).

7. Zeng, T. et al. Zeolite-Based Memristive Synapse with Ultralow Sub-10-fJ Energy Consumption for Neuromorphic Computation. *Small* **17**, 2006662 (2021).

8. Zhang, Q. J. et al. Ternary flexible electro-resistive memory device based on small molecules. Chem. Asian J. 11, 1624-1630 (2016).

9. Yu, A-D. et al. Tunable Electrical Memory Characteristics Using Polyimide:Polycyclic Aromatic Compound Blends on Flexible Substrates. ACS Appl. Mater. Interfaces 5, 4921-4929 (2013).

10. Wu, H-C. et al. Donor–acceptor conjugated polymers of arylene vinylene with pendent phenanthro[9,10-d]imidazole for high-performance flexible resistor-type memory ap-plications. Polym. Chem. 4, 5261-5269 (2013).

11. Zhang, B. et al. 90% yield production of polymer nano-memristor for in-memory computing. Nat. Commun. 12, 1984 (2021).

12. Yin, Y-H. et al. Direct photopolymerization and lithography of multilayer conjugated polymer nanofilms for high performance memristors. J. Mater. Chem. C. 6, 11162-11169 (2018).

13. Ren, Y. et al. Iridium-based polymer for memristive devices with integrated logic and arithmetic applications. J. Mater. Chem. C. 8, 16845 (2020).

14. Liu, G. et al. Resistance-switchable graphene oxide-polymer nanocomposites for molecular electronics. ChemElectroChem 1, 514-519 (2014).

15. Lu, Y. et al. Stretchable and twistable resistive switching memory with information storage and computing functionalities. Adv. Mater. Technol. 6, 2000810 (2021).

16. Zhou, Y. et al. Ultra-flexible nonvolatile memory based on donor-acceptor

diketopyrrolopyrrole polymer blends. Scientific Reports 5, 10683 (2015).

17. Seung, H.-M. et al. Flexible conductive-bridging random-access memory cell vertically stacked with top Ag electrode, PEO, PVK, and bottom Pt electrode. Nanotechnology. 25, 435204 (2014).

18. Shih, C-C. et al. Multi-state memristive behavior in a light-emitting electrochemical cell. J. Mater. Chem. C 5, 11421-11428 (2017).

19. Shi, R. et al. Fully solution-processed transparent nonvolatile and volatile multifunctional memory devices from conductive polymer and graphene oxide. Adv. Electron. Mater. 3, 1700135 (2017).

20. Yoo, D. et al. Vertically stacked microscale organic nonvolatile memory devices toward three-dimensional high integration. Org. Electron. 21, 198-202 (2015).

21. Wu, H. C. et al. Well-defined star-shaped donor-acceptor conjugated molecules for organic resistive memory devices. Chem. Commun. 51, 14179-14182 (2015).

22. Shih, C-C. et al. Transparent deoxyribonucleic acid substrate with high mechanical strength for flexible and biocompatible organic resistive memory devices. Chem. Commun. 52, 13463-13466 (2016).

23. Wu, W. et al. Biodegradable skin-inspired nonvolatile resistive switching memory based on gold nanoparticles embedded alkali lignin. Org. Electron. 59, 382-388 (2018).

24. Asadi, K., Li, M., Stingelin, N., Blom, P. W. M. & de Leeuw, D. M. Crossbar memory array of organic bistable rectifying diodes for nonvolatile data storage. Appl. Phys. Lett. 97, 193308 (2010).

25. Liu, Z-D. et al. Flexible metal-free memory electronic made of π-conjugation-interrupted hyperbranched polymer switch and reduced graphene oxide electrodes. Macromol. Mater. Eng. 305, 2000050 (2020).

26. Tseng, R. J. et al. Digital memory device based on tobacco mosaic virus conjugated with nanoparticles. Nat. Nanotech. 1, 72-77 (2006).

27. Liao, Q. et al. Electronic synapses mimicked in bilayer organic-inorganic heterojunction based memristor. Org. Electron. 90, 106062 (2021).

28. Kim, T. W. et al. A direct metal transfer method for cross-bar type polymer non-volatile memory applications. Nanotechnology 19, 405201 (2008).

29. Ling, Q.-D. et al. Polymer memories: Bistable electrical switching and device performance. Polymer 48, 5182-5201 (2007).

30. Mao H-W. et al. Control of resistive switching voltage by nanoparticle decorated wrinkle interface. Adv. Electron. Mater. 5, 1800503 (2019).

31. Huang, R. et al. Resistive switching in organic memory device based on Parylene-C with highly compatible process for high-density and low-cost memory applications. IEEE Trans Electron. Dev. 59, 3578-3582 (2012).

32. Wang, J-T. et al. High-performance stretchable resistive memories using donor–acceptor block copolymers with fluorene rods and pendent isoindigo coils. NPG Asia Mater. 8, e298 (2016).

33. Zhou, L. et al. A UV damage-sensing nociceptive device for bionic applications. Nanoscale 12, 1484-1494 (2020).

34. Liu, X.-H. et al. Fabrication of a 256-bits organic memory by soft x-ray lithography. Chin. Phys. B 19, 057204 (2010).

35. Ahn B. et al. Tunable film morphologies of brush−linear diblock copolymer bearing difluorene moieties yield a variety of digital memory properties. ACS Macro Lett. 2, 555-560, (2013).

36. Kuang, Y.-B. et al. Flexible single-component-polymer resistive memory for ultrafast and highly compatible nonvolatile memory applications. IEEE Electron. Dev. Lett. 31, 758-760 (2010).

37. Wang, L. et al. Long-term homeostatic properties complementary to Hebbian rules in CuPc-based multifunctional memristor. Scientific Reports 6, 35273 (2016).

38. Portney, N. G. et al. Microscale memory characteristics of virus-quantum dot hybrids. Appl. Phys. Lett. 90, 214104 (2007).

39. Prakash, A. et al. Polymer memory device based on conjugated polymer and gold nanoparticles. J. Appl. Phys. 100, 054309 (2006).

40. Mao, J.-Y. et al. A bio-inspired electronic synapse using solution processable organic small molecule. J. Mater. Chem. C 7, 1491-1501 (2019).

41. Jameson, J. R. et al. Quantized conductance in Ag/GeS_2/W conductive-bridge memory cells. IEEE Electron Device Lett 33, 257-259, (2012).

42. Tappertzhofen, S., Valov, I. & Waser, R. Quantum conductance and switching kinetics of AgI-based microcrossbar cells. *Nanotechnology* **23**, 145703, (2012).

43. Terabe, K. et al. Quantized conductance atomic switch. *Nature* **433**, 47–50 (2005).

44. Jiang, L. et al. Conductance quantization in an AgInSbTe-based memristor at nanosecond scale. *Appl. Phys. Lett* **109**, 153306, (2016).

45. Liu, D., Cheng, H., Zhu, X., Wang, G. & Wang, N. Analog memristors based on thickening/thinning of Ag nanofilaments in amorphous manganite thin films. *ACS Appl Mater Interfaces* **5**, 11258-11264, (2013).

46. Gao, S. et al. Resistive switching and conductance quantization in Ag/SiO_2/indium tin oxide resistive memories. *Appl. Phys. Lett* **105**, 063504, (2014).

47. Gao, S. et al. Conductance quantization in a Ag filament-based polymer resistive memory. *Nanotechnology* **24**, 335201, (2013).

48. Tappertzhofen, S. et al. Modeling of quantized conductance effects in electrochemical

metallization Cells. *IEEE Trans. Nanotechnol* **14**, 505-512, (2015).

49. Tsuruoka, T., Hasegawa, T., Terabe, K. & Aono, M. Conductance quantization and synaptic behavior in a Ta_2O_5-based atomic switch. *Nanotechnology* **23**, 435705, (2012).

50. Krishnan, K., Muruganathan, M., Tsuruoka, T., Mizuta, H. & Aono, M. Highly reproducible and regulated conductance quantization in a polymer-based atomic switch. *Adv. Funct. Mater* **27**, 1605104, (2017).

51. Wang, Z-R. et al. Memristors with diffusive dynamics as synaptic emulators for neuromorphic computing. *Nat. Mater*. **16**, 101-108 (2017).

52. Jiang, H. et al. A novel true random number generator based on a stochastic diffusive memristor. Nat. Commun. **8**, 882 (2017).

53. Dudek, S. et al. Homosynaptic long-term depression in area CA1 of hippocampus and effects of N-methyl-D-aspartate receptor blockade. *Proc. Natl. Acad. Sci.* **89**, 4363-4367 (1992).

54. Wang, Z. et al. Toward a generalized Bienenstock-Cooper-Munro rule for spatiotemporal learning via triplet-STDP in memristive devices. *Nat. Commun*. **11**, 1510 (2020).

55. Demin, V. et al. Necessary conditions for STDP-based pattern recognition learning in a memristive spiking neural network. *Neural Networks* **134**, 64-75 (2021).

56. Chen, L. & Aihara, K. Chaotic simulated annealing by a neural network model with transient chaos. *Neural Networks* **8**, 915-930 (1995).
